# Supplementary material for: A Method for In Situ Reverse Genetic Analysis of Proteins Involved mtDNA Replication
Source: Cells. 2022 Jul 11;11(14):2168. doi: 10.3390/cells11142168 (PMC9316749; doi:10.3390/cells11142168)
Supplement: Supplementary file 1 [file cells-11-02168-s001.zip › cells-1787828-Supplementary.pdf]

Supplementary Materials

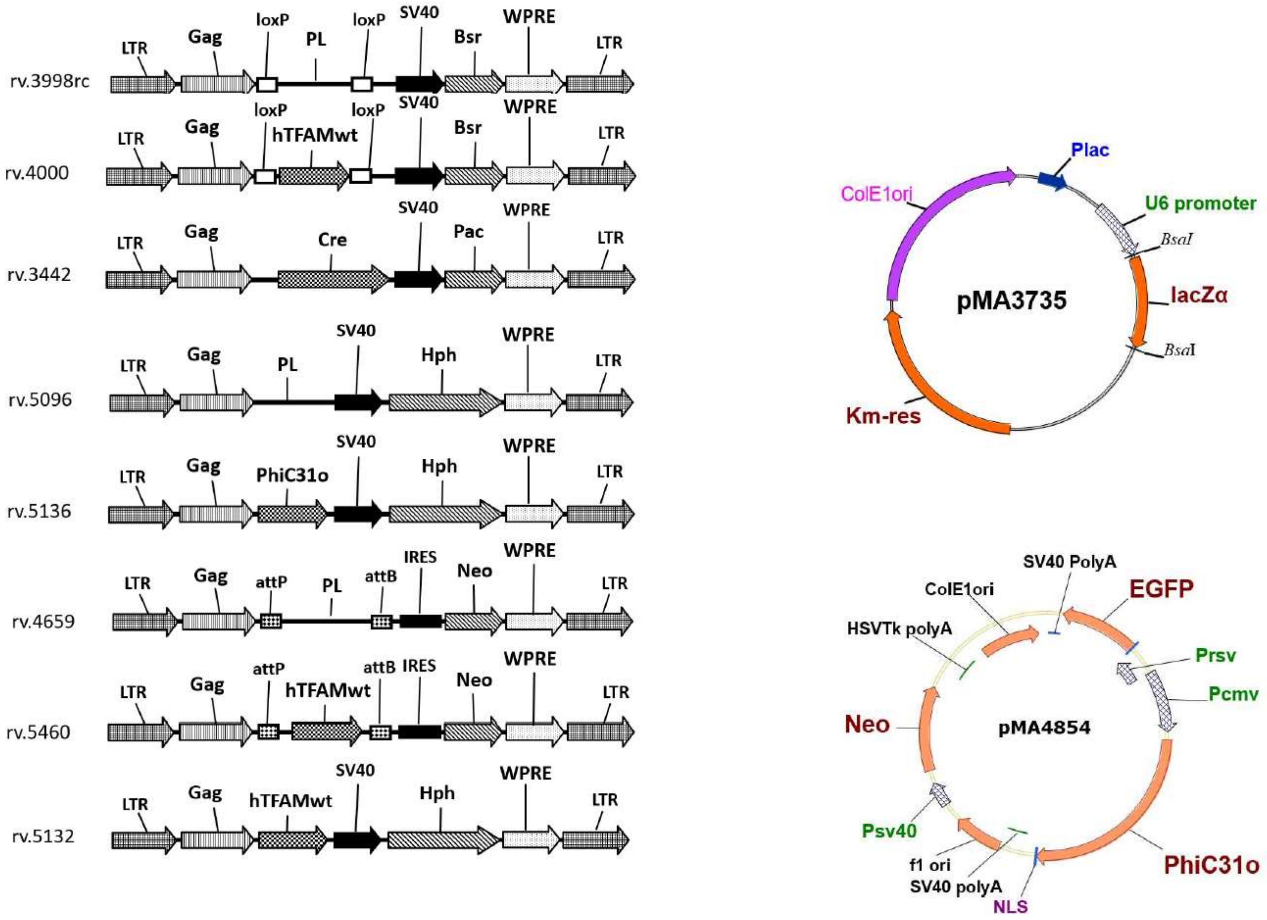

Figure S1. Maps of vectors used in this study. Designations: attP, attB, recombination sites for PhiC31 recombinase; Bsr, Blasticidin resistance gene; Cre, gene encoding the bacteriophage P1 Cre recombinase; ColE1ori, origin of bacterial replication from ColE1 plasmid; Gag, truncated retroviral Gag protein; f1 ori, replication origin of the *E. coli* bacteriophage f1; Hph, hygromycin phosphotransferase, hygromycin resistance gene; hTFAMwt, wild-type hTFAM; HSVTk PolyA, termination/polyadenylation signal from Herpers Simplex Virus thymidine kinase gene IRES, internal ribosome entry site; Km-res, bacterial kanamycin resistance gene; lacZα, α-fragment of the *E. coli* β-galactosidase gene; loxP, recombination sites for Cre recombinase; LTR, retroviral long terminal repeat; mTFAM, murine TFAM; Neo, G418 and kanamycin resistance gene; NLS, nuclear localization sequence; Pac, puromycin resistance gene; Pcmv, cytomegalovirus immediate-early promoter; PhiC31o, optimized PhiC31 recombinase gene; PL, polylinker; Plac, lac promoter; Prsv, promoter of the Rouse Sarcoma Virus, SV40, promoter of the SV40 virus; SV40 polyA, termination/polyadenylation signal from SV40 virus; WPRE, woodchuck hepatitis virus posttranscriptional regulatory element.

The vectors were deposited with Addgene. The utility of the vectors is as follows:

rv.3998rc (Addgene#184851). A general-purpose retrovirus vector encoding blasticidin resistance. Polylinker is flanked by loxP sites. Used to generate rv.4000 for the delivery of Cre-excisable wt hTFAM gene (see Figure 1B).

rv.4000 (Addgene#184857). A retrovirus encoding an excisable wt hTFAM gene flanked by loxP sites for Cre recombinase and blasticidin resistance. Used to engineer 143B#6 cell line (Figure 1B, C, G)

rv.3442 (Addgene#1848521). A retrovirus encoding Cre recombinase and puromycin resistance. Used to deliver Cre recombinase to effect wt hTFAM excising in 143B#6 cells (Figure 1C, G).

rv.5096 (Addgene#184850). A general-purpose retrovirus vector encoding hygromycin resistance. Used to generate rv.5136 for the delivery of PhiC31 recombinase.

rv.5136 (Addgene#184853). A retrovirus encoding PhiC31 recombinase and hygromycin resistance. Derived from rv.5091. Used to deliver PhiC31o recombinase to effect TFAMvar excision (Figure 1C).

rv.4659 (Addgene#184854). A general-purpose retrovirus vector encoding G418 resistance. Contains a polylinker flanked by attP and attB sites for PhiC31 recombinase. Used to generate retroviral constructs encoding PhiC31-excisable TFAM derivatives.

rv.5460 (Addgene#184855). A retrovirus encoding an excisable wt hTFAM flanked by attP and attB sites for Cre recombinase and G418 resistance. Derived from rv.4659. Used to swap wt hTFAM for wt hTFAM in 143B#6 cells (control experiment).

rv.5132 (Addgene#184858). A retrovirus encoding a wt hTFAM and hygromycin resistance. A derivative of rv.5091. Used to complement mtDNA replication and transcription defects in cells expressing coelTFAM (Figure 3).

pMA3735 (Addgene#184849). A plasmid vector for expressing sgRNA constructs. Oligos corresponding to gRNA are annealed and cloned between BsaI sites of this vector. Recombinant E. coli colonies are white on plates supplemented with kanamycin, IPTF, and Xgal.

pMA4854 (Addgene#184849). A plasmid encoding EGFP and PhiC31o recombinase. Used to transiently deliver PhiC31o recombinase (e.g., Figure 2D).

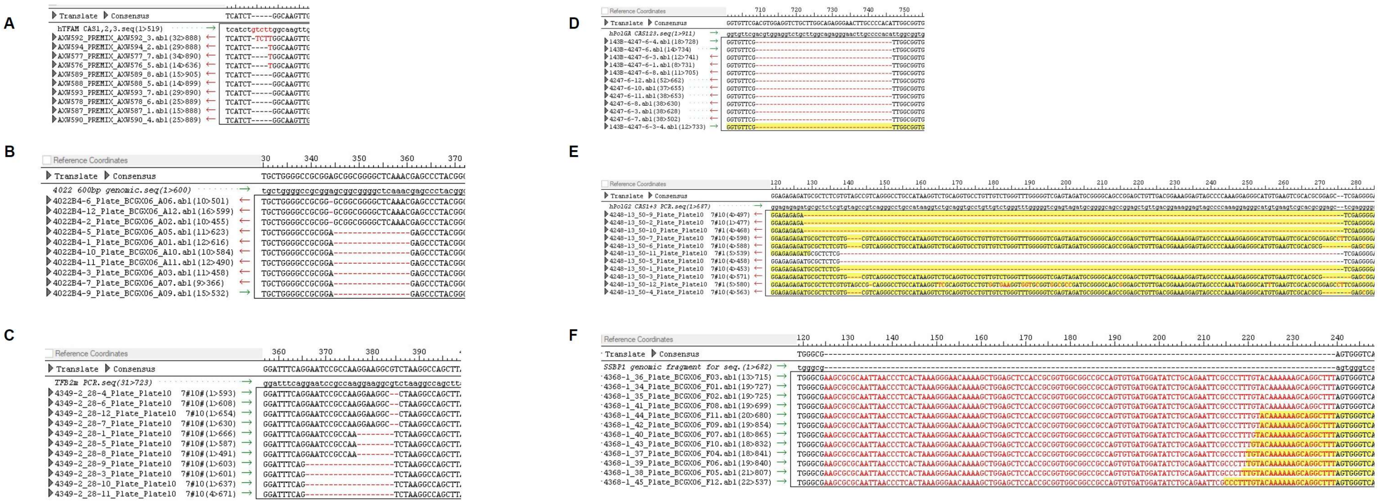

Figure S2. Alignments of the sequenced genomic loci in 143B cells. TFAM (A), POLRMT (B), TFB2M (C), POLG1 (D), POLG2 (E), and SSBP1(F) loci were targeted with gRNAs (STAR Methods) expressed from pMA3735 vector (Supplementary figure S2). Targeted cells were cloned, the targeted locus in individual clones was PCR-amplified, fragments were cloned in pBluescriptII SK+, and plasmid DNA from the resulting bacterial clones was Sanger sequenced.

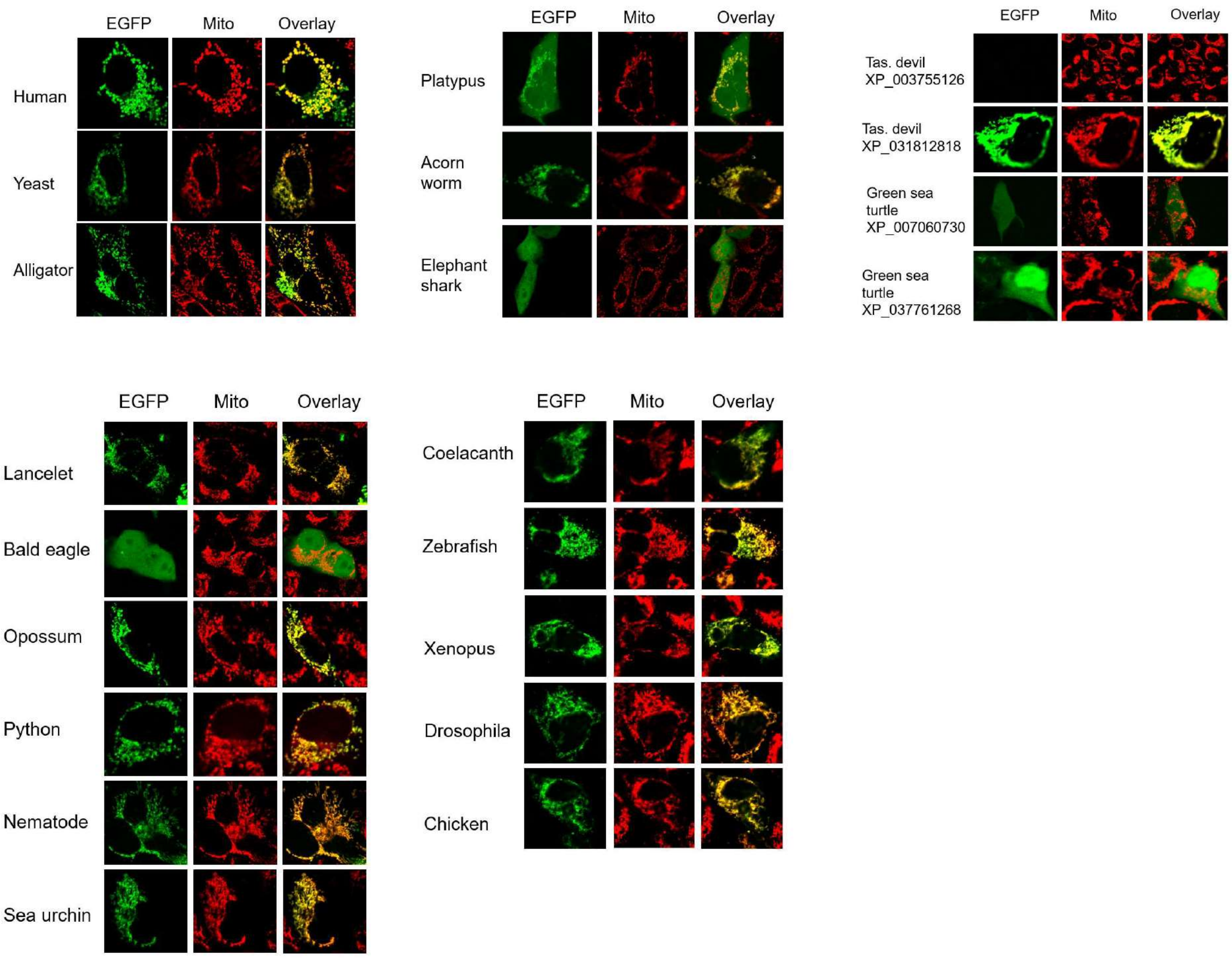

Figure S3. oTFAM MTSs are functional in human cells. oTFAM MTSs were amplified by PCR, fused to EGFP, transfected into 143B cells under the control of CMV promoter, and imaged with Nicon A1R confocal system after staining mitochondrial with MitoTracker Red CMXROS.

Table S1. Oligonucleotides used in this study.

Purpose	Name	Sequence	Amplicon, bp
**Genotyping**			
Genotyping of the ρ^0 state in human cells	hMitF	AATGTCTGCACAGCCACTTTCCAC	Mit=901
	hMitR	TCGTAGTGTTCTGGCGAGCAGTTT	
	hNucF	AGAATGTGGCAGTGGAGATG	Nuc=371
	hNucR	GCGAGTGCTGCCCCGAC	
Genotyping of hTFAM excision in 143B#6 cells	ExF2	CGCCTCAATCCTCCCTTTATC	WT=408
	ExR2	CACACCTGGTTGCTGACTAA	Excised=251
	ExInt2	GCATCTGGGTTCTGAGCTTTA	
Genotyping Cre recombinase	Cre-3	CTGCCACGACCAAGTGACAGC	314
	Cre-4	ACCTGCGGTGCTAACCAGCG	
Genotyping PhiC31 recombinase	PhiC31F1	AAGCGCCAACGAGGATAAG	308
	PhiC31R1	TCACGTTGCCCTGTCTAAAG	
**RT-qPCR of transcripts**			
hHPRT	hHPRTf	CGAGATGTGATGAAGGAGATGG	N/A
	hHPRTr	TTGATGTAATCCAGCAGGTCAG	
hMT-ND1	hND1F	GAAGTCACCCTAGCCATCATTC	N/A
	hND1R	GCAGGAGTAATCAGAGGTGTTC	
hMT-ND6	hND6F	CCACACCGCTAACAATCAATAC	N/A
	hND6R	GTTTCTGTTGAGTGTGGGTTTAG	
hMT-RNR2 (16S)	h16Sf	GAAACCAGACGAGCTACCTAAG	N/A
	h16Sr	GGTTTGTCGCCTCTACCTATAAA	
hMT-CO1	hCox1F	CTAGCAGGTGTCTCCTCTATCT	N/A
	hCox1R	GGCGTTTGGTATTGGGTTATG	
mtDNA copy number determination by ddPCR, human cells	NucF	AACTTGTAAGTGGTAGTGCATAGA	N/A
	NucR	GTAGGAGGACATTTGAGGAGTG	
	NucProbe	FAM-TCAGGCAGACTGACACTAGAGTTCACA-BHQ1	
	MitF	CTGATCAGGGTGAGCATCAAA	
	MitR	GAATGATGGCTAGGGTGACTTC	
	MitProbe	Hex-TGCGAGCAGTAGCCCAAACAATCT-BHQ1	
**Genotyping of theTFAM orthologs (oTFAM) cDNAs**			
hTFAM	2F	GTTGGAGGGAACTTCCTGATT	349
	2R	TGCTGAATATATAATTCCTTTTCAGAGT	
Cat TFAM	CatF	AGAAACCGTTGACGAGCTATG	387
	CatR	CCTGTGAAGTCCCGTCTTTATG	
Pig TFAM	PigF	CCTATGTACCTCGCTGGTTTAG	632
	PigR	GCACTCCTCAGTGTCTTTCTT	
Manatee TFAM	ManF	CAAAGAGGAAATCAACCGCATC	322
	ManR	GGACTTCATCTCGTTGTCGTAG	
Pika TFAM	PikaF	GTGGAATGGGAAGCCTACAA	349

	PikaR	GTTCTTCCCAGGACTTCATCTC	
Armadillo-like TFAM	Arm-lF	CCAGAAGATGGATCAGCAGC	455
	Arm-lR	CCAGTTCAGGATCAGTGTCTTC	
Hedgehog TFAM	HedgeF	ACAGCGAGCTGATCAAGAAG	281
	HedgeR	AAACCGCTCGGCGATAAA	
Elephant TFAM	ElephF	GTGGCAGCAGACTGAGATT	278
	ElephR	GATGCGGTTCACTTCTTCTTTG	
Elephant shrew TFAM	ShrewF	CTGAGCAGCTTCCTGAGATT	578
	ShrewR	CAGTCGACGTTCTCGTTCTT	
Bat TFAM	BatF	TTAGCTCTACCCTCTCATCCTAC	329
	BatR	CAGTGTCAGTTCCCGTTTCT	
Murine TFAM	MouF	GGAGCTACCAGAAGCAGAAA	340
	MouR	CTGAATATATGCCTGCTTTTCCTC	
Coelacanth TFAM	CoelF	CAAGCCTAAACGGCCTAGAA	229
	CoelR	ATCCTCCCTCCCTATGTCTATC	
Zebrafish TFAM	ZfF	GGAGGCCAAGGAGCAATATAA	262
	ZfR	GCGATTCCAGTCATCTCTTAGT	
Frog TFAM	XlF	CACCATCTGACTACCCAAAGAG	381
	XlR	CCTGGAAGTGCTCAGACATAAA	
Armadillo TFAM	ArmF	CCCTCGGTGGTACTCTTCT	547
	ArmR	CTGTTCTTCCCAGGACTTCATC	
Tasmanian devil TFAM	TasF	CTGTGGACTGGAATGGTTCAT	388
	TasR	GTTATACCCTGAGCGAGGTTTC	
Opossum TFAM	OpF	CTCTGACCAGCTACATCAGATTC	422
	OpR	GCTCAGGTTCTTCCACTCTTT	
Platypus TFAM	PlatF	CGACCTCTGACAGCTTATCTTC	296
	PlatR	GTGAGTTCCCGCTTCTTCTT	
Chicken TFAM	ChickF	CCAGAAGCAAGTGTACGAAGAG	379
	ChickR	CGCCTCCCAGGATTTCATTT	
Turtle TFAM	TurtF	TCCGAGAAGCAGACCTATGA	396
	TurtR	CACGTCCACCATCTGTTCTT	
Bald eagle TFAM	EagleF	AGAAACAGGTGTACGAGGAAAG	331
	EagleR	TCGGCCAGTTGCAGATAAG	
Python TFAM cDNA	PythF	CTGACTCCTTACCTGCACTTTC	484
	PythR	GCTCGTTGTCGTATCTGATCTT	
Elephant shark TFAM	SharkF	GAGGGCAAGCTGAAGTACAA	339
	SharkR	GCTCTTGATCTCGTTCTGGTATC	
Alligator TFAM	AlligF	ACCTCCTAGCGCTCCTAAA	514
	AlligR	CTTCATCTCGTTGGCGTATCT	
Drosophila TFAM	DmF	AGCGGGACCAACAAATCTAC	369
	DmR	GGATCATCTTCTCCTCCCAAAC	
Lancelet TFAM	BrachF	CACTGAGAAGCAGCCCTATATC	365
	BrachR	GCATTTCGGCTTCGTATTTCTC	
Sea urchin TFAM	UrchinF	CGACCACTCACATCCTTCTTC	323
	UrchinR	GGAGGTCGTTTCGGCTTATT	
Acorn worm TFAM	SacF	ATTTCCACTACTCTGCGGTATG	448
	SacR	CTTTCTGGCGTCCCTTACTATC	

Nematode TFAM	CelF	CCGATCTGATGAAGGAGTTGAG	408
	CelR	GCACTACATGGTACTCGTCTTT	
Yeast abf2 cDNA	Abf2F	CCAAGAGAACGCAGCTTAGAA	462
	Abf2R	TGAGAGGGTAGCGAGCATTA	
Genotyping of oTFAM excision in GeneSwapped cells (ΔPhiC31)			
Main	F	ACCTACCCGAGTCGGACTTT	
	R1	GTTATTGCTTGGGATGTACTTGG	Ex=370
	Mouse_intF	CAGCTGATGGGTATGGAGAAG	WT=505/1241
	Armadillo-like_intF	CTGCGGCAATAGACTGAGAT	WT=784/1222
	Pig_intF	CCTATGTACCTCGCTGGTTTAG	WT=749/1222
	Frog_intF	GCAAGTCCGTGAGGAAGAAG	WT=605/1483
	Human_intR	TGCATCTGGGTTCTGAGCTTTA	WT=547/1216
	Elephant shrew_intR	GCTCTTGGATGCGGTTGATTTC	WT=725/1213
	Coelacanth_intR	GATCCGGAATTGTCGCTAAA	WT=573/1435
	Zebrafish_intR	GCTGGGCAATCTTTCGCATAAC	WT=617/1378
	Pica_intR	TTGTAGGCTTCCCATTCCAC	WT=699/1222
	Elephant_intR	CACGAAGCTCAGAGGGAATC	WT=475/1228
	Hedgehog_intR	TAGGCGTCCTCGTACAGCTT	WT=675/1228
Alternative	F	ACCTACCCGAGTCGGACTTT	
	R2	GAGGAACTGCTTCCTTCACG	Ex=606
	Cat_intR	ATTTGCCCAGAGCAGACAG	WT=416/1222
	Manatee_intR	GCTCTTGGATGCGGTTGATTTC	WT=725/1222
	Bat_intR	AGATTTCCCCAATGCTGAC	WT=419/1219
**Genotyping of TFAM chimeras. Chimera-specific primers**			
Human-Tasmanian devil	H-TdF	GCGGAGTGGCAGGTATATAAAG	304
	H-TdR	CTTCCGCCAGTTGAAGATAGAC	
Tasmanian devil-human	Td-hF	GAACTGCCACAAAGCAAGAAG	472
	Td-hR	AACACTCCTCAGCACCATATTT	
Human-opossum	H-opF	GCGGAGTGGCAGGTATATAAAG	354
	H-opR	CATCTTCTCTTCCCAGCTCTTG	
Opossum-human	Op-hF	CCAAGAACAGGTGTCCAAGTA	300
	Op-hR	CGAGTTTCGTCCTCTTTAGCA	
Armadillo-human	Ar-hF	GCCTACAAAGAGGCCATCAA	359
	Ar-hR	TCCTTTCGTCCAACTTCAATCA	
Human-armadillo	H-arF	GCGGAGTGGCAGGTATATAAAG	339
	H-arR	GGACTTCATCTCGTTGTCGTATC	
Alligator-human	Al-hF	TGACCGTGAAGAAGCAGAAC	543
	Al-hR	AACACTCCTCAGCACCATATTT	
Human-alligator	H-alF	GTTGGAGGGAACTTCCTGATT	389
	H-alR	CTTCATCTCGTTGGCGTATCT	
Lancelet-human	Lan-hF	CACTGAGAAGCAGCCCTATATC	351
	Lan-hR	CGAGTTTCGTCCTCTTTAGCA	
Human-lancelet	H-lanF	GTTGGAGGGAACTTCCTGATT	384
	H-lanR	GCATTTCGGCTTCGTATTTCTC	
Drosophila-human	Dm-hF	AGCGGGACCAACAAATCTAC	376
	Dm-hR	TCCTTTCGTCCAACTTCAATCA	

Human-drosophila	H-dmF	GCGGAGTGGCAGGTATATAAAG	360
	H-dmR	CTTCTCCTCCCAAACGGAAAT	
Platypus-human	Plat-hF	GTGTATCGGAAAGCAGCAAAC	450
	Plat-hR	TTAACACTCCTCAGCACCATATT	
Human-platypus	H-platF	GCGGAGTGGCAGGTATATAAAG	373
	H-platR	CTTTCCGGCCTATGTCAATCA	
Python-human	Pyth-hF	GTGGGACATCTTCAGAGAACAG	422
	Pyth-hR	AACACTCCTCAGCACCATATTT	
Human-python	H-pythF	GTTGGAGGGAACTTCCTGATT	384
	H-pythR	GCTCGTTGTCGTATCTGATCTT	
Shark-human	Sh-hF	GAGGGCAAGCTGAAGTACAA	427
	Sh-hR	AACACTCCTCAGCACCATATTT	
Human-shark	H-shF	GCGGAGTGGCAGGTATATAAAG	339
	H-shR	GCTCTTGATCTCGTTCTGGTATC	
Acorn worm-human	W-hF	GCCCTCAAACTCTCCAAACA	635
	W-hR	GTGCGACGTAGAAGATCCTTTC	
Human-acorn worm	H-wF	GCGGAGTGGCAGGTATATAAAG	229
	H-wR	CTTTCTGGCGTCCCTTACTATC	
Human-turtle	H-tF	GCGGAGTGGCAGGTATATAAAG	351
	H-tR	CTGTTCTTCCCAGGACTTCATC	
Turtle-Human	T-hF	TCCGAGAAGCAGACCTATGA	462
	T-hR	TTAACACTCCTCAGCACCATATT	
Human-sea urchin	H-suF	GCGGAGTGGCAGGTATATAAAG	246
	H-suR	CTGGGCTTGAATCTCTTCTCTG	
Sea urchin-h	Su-hF	CGACCACTCACATCCTTCTTC	586
	Su-hR	AACACTCCTCAGCACCATATTT	
Human-chicken	H-chF	GCGGAGTGGCAGGTATATAAAG	500
	H-chR	GCCTCGAGTCATTCTTCTGATT	
Chicken-human	Ch-hF	CCAGAAGCAAGTGTACGAAGAG	420
	Ch-hR	GTGCGACGTAGAAGATCCTTTC	
**Genotyping of TFAM chimera excision in GeneSwapped cells (F+R1 and F+R3)**			
F	ACCTACCCGAGTCGGACTTT		
R1	GTTATTGCTTGGGATGTACTTGG		Ex=370
R3	CACACCGGCCTTATTCCAAG		Ex=448
Armadillo-human			WT=1213
Human-armadillo			WT=1222
MTS-Tas.devil			WT=1229
Tas. Devil-human			WT=1291
MTS-Tas.devil-human			WT=1223
Human-tas.devil			WT=1228
Opossum-human			WT=1249
Human-opossum			WT=1228
MTS-Platypus			WT=1186
Platypus-human			WT=1264
MTS-Platypus-human			WT=1232
Human-platypus			WT=1210

MTS-Turtle		WT=1309
Turtle-human		WT=1165
MTS-turtle-human		WT=1229
Human-turtle		WT=1333
Python-human		WT=1198
Human-python		WT=1234
MTS-shark		WT=1276
Shark-human		WT=1102
Human-shark		WT=1306
Drosophila-human		WT=1219
Human-drosophila		WT=n.a.
Lancelet-human		WT=1216
Human-lancelet		WT=1321
Sea urchin-human		WT=1228
Human-sea urchin		WT=1180

Table S2. Sequences of TFAM variants used in this study.

Murine TFAM wt	Atggcgctgttccggggaatgtggagcgtgctaaaagcactggggcgcacgggggtcgagatgtgcgcgggctgcgggggtcgcatccc<br>ctcgtctatcagtcttgtctgtattccgaagtgtttttccagcatgggtagctatccaaagaaacctatgagttcatacctccgattttccaca<br>gaacagctacccaaatttaaagctaaacacccagatgcaaaactttcagaattggttaggaaaattgcagccctgtggagggagctacc<br>agaagcagaaaaaaaggtttatgaagctgattttaaagctgagtggaaagcatacaaagaagctgtgagcaagtataaagagcagcta<br>actccaagtcagctgatgggtatggagaaggaggcccggcagagacggttaaaaaagaaagcactggtaaagagaagagaattaatt<br>ttgcttggaaaaccaaaaagacctcgttcagcatataacatttatgtatctgaaagcttccaggaggcaaaggatgattcggctcaggga<br>aaattgaagcttgtaaatgaggcttggaaaaatctgtctcctgaggaaaagcaggcatatattcagcttgctaaagatgataggattcgtt<br>acgacaatgaaatgaagtcttgggaagagcagatggctgaagttggacgaagtgatctcatccgtcgaagtgtgaaacgatccggaga<br>catctctgagcattaa
Human TFAM wt	Atggcgtttctccgaagcatgtggggcgtgctgagtgccctgggaaggtctggagcagagctgtgcaccggctgtggaagtcgactgcgc<br>tcccccttcagttttgtgtatttaccgaggtggttttcatctgtcttggcaagttgtccaaagaaacctgtaagttcttaccttcgattttctaa<br>agaacaactacccatatttaaagctcagaacccagatgcaaaaactacagaactaattagaagaattgcccagcgttggagggaacttc<br>ctgattcaaagaaaaaaatatatcaagatgcttatagggcggagtggcaggtatataaagaagagataagcagatttaaagaacagct<br>aactccaagtcagattatgtctttggaaaaagaaatcatggacaaacatttaaaaaggaaagctatgacaaaaaaaaaagagttaaca<br>ctgcttggaaaaccaaaaagacctcgttcagcttataacgtttatgtagctgaaagattccaagaagctaagggtgattcaccgcaggaa<br>aagctgaagactgtaaaggaaaactggaaaaatctgtctgactctgaaaaggaattatatattcagcatgctaaagaggacgaaactcg<br>ttatcataatgaaatgaagtcttgggaagaacaaatgattgaagttggacgaaaggatcttctacgtcgcacaataaagaaacaacgaa<br>aatatggtgctgaggagtgttaa
Cat TFAM	atggcactgtttcggggagtgtggagcgtactgtctgctctgggcaaatccggagctgacctgtgtgctggttgcggttctcggcttaggag<br>cccattcagctttgcctatgttccacggtgcttcagttctactgtgaatagctatcccaagaaaccgttgacgagctatgtccggttctccaa<br>agaacagctgcctatcttcaaggcccagaatccagacgccaagaactccgaacttatccgtaaaatcgcacaactgtggagagaactcc<br>ctgattccgagaagaaaatctatgaggatgcgtatcgagcagattggcaagcctacaaggaagagattaaccgcatacaagaacagct<br>gacaccctcccagattgtcagtttggagaaggagattcagcagaagcgcctcaagaagaaagctctgatcaagaaacgcgaactcacc<br>atgttgggcaagcccaagaggcctagatcagcctacaacatctacatagcagagaggtttcaggagcataaagacgggacttcacaggt<br>gaaactgaaaaccatcaacgaaaactggaagaatctctcaagttctcagaagcaagtgtacattcagctggccaatgacgacaagatta<br>ggtactacaacgagatgaaaagctgggaggaacagatgctggaggttggcagaaatgatcttctgcgaagaacagtgaaacaccaggc<br>gaagaatgggatagaggagtacggatcctag
Pig TFAM	Atggcactgcttaggggcgtgtggggcgtgctgagcgccctgggcaaatctggggcagacttgtgtgcagtatgcggtagccgactgcgc<br>tctcccttcagctttgcctatgtacctcgctggtttagcagtaccctgagcgggtttccgaagaagccaatgacgagctacgtcaggttctc<br>aaaagaacagctgcccattttcaaagctcagaaccctgatgccaagaactcagagctgatcaagaaaattgctgagctgtggcgagaac<br>tgcccgatagtgagaagaagatttatgaggatgcgtatagagcagactggcaagtgtacaaagaggaagttaaccggatacaggaaca<br>gctcacaccttcccaaatggtttccctggaaaaggagatcatgcagaagagattgaagaagaaggcccttatcaagaagagggaactg<br>acaatgttgggaaaacccaaaagaccacggtccgcttacaacatattcatcgccgaaaggtttcaggaagccaaggatggtccatcaca<br>ggtgaaactcaagaccatcaatgagaactggaagaatctctctagctcccagaaacaggtgtatatccaacttgctgaggatgacaaag<br>tccggtactacaatgagatgaaaagctgggaagagcagatggtggaggtcggaagaaatgacctcattcgtcgctccatgaaacactct<br>gcgaagaaagacactgaggagtgcggatcctag

Manatee TFAM	Atggctctgcttagaggcgtttggggagtgctgtctgccctgggaaaatctggcgccgaactgtgtgccggctgtggcagcagactgagattccctctgagcttcgtgtgcgtgcccagatggtttagcagcaccctgggcagctaccccaagaagcctctgagcagctacctgcggtttagcaccgagcagctgcccatcttcaaggcccagaatcctgacgccaagaacagcgagctgatcaagaaaatcgcccagctgtggcgcgagctgcccgattctgagaagaaggtgtacgaggacgcctacaaggccgactggcaggcctacaaagaggaaatcaaccgcatccaagagcagctgacccctagccagatcgtgtccatggaaaaagaaatcatgcagaagcggctgaagcggaaggccctgatcaaaaagcgggaactgaccatgctgggcaagcccaagaggcctagaagcgcctacaacatctttatcgccgagagctttcaagaggccaaggacgccagctctcaggccaagctgaaaaccgtgaacgagaactggaagaacctgagcaccagccagaaacaggtgtacatccagctggccaaggatgacaagatccgctacgacaacgagatgaagtcctgggaagcccagatgatcgaagtgggcagaaacgacctgatccggcggaagatgaagcagcggaccaaggatggcaccgaggaatgtgtcgactga
Pika TFAM	Atggctctgctgagaggcgtttggggcgtgctgagaacactgggaaaatctggcgccgatctgtgtgccggctgtggcagtagactgagaagccccttcagcttcagctacgtgcccaagtggtgcagcagcaccttcagcagctaccccaagaaacccatgaccagctacctgcggttcagcaaagagcagctgcccatcttcaaggcccagaatcctgacgccaagaacagcgagctgatcaagaaaatcgccgaagtgtggcgcgagctgcccgactctgagaagaaagtgtacgaggacgcctaccgcgtggaatgggaagcctacaaagaggaaatcagccgcatccaagagcacctgacacctagccagctgctgagcatggaaaaagaaatcatgcagaagcggctgcggaagaaggccctgatcaaaaagcgggaactgaccatgctgggcaagcccaagaggcctagaagcgcctacaacatcttcgtgtccgagagctttcaagaggccaaggacggaagcagccaggccaaactgaaggccgtgaacgacaactggaagaacctgcctaccagccagaaacaggtgtacatccagctggccaaggatgacaagatccgctacgacaacgagatgaagtcctgggaagaacagatgatcgaagtgggcagaagcgacctgatccggcggaagatgatgcaccagagcaaggacggcaccgaggaaagagtcgactga
Armadillo TFAM-like	Atggccctgtttagaggcgtttggggcctgctgtctagcctgggaaaatctggcgcccagctgtactccggctgcggcaatagactgagattcagcctgagcttcgcccggaccagaagatggatcagcagcacactgggcagctaccccaagaagcctctgagcagctacctgcggttcaccaaagagcagctgcccatcatcaaggcccagaatcctgacgccaagattcccgagatcatccagaaaatcgcccagcagtggcgcgagctgcctgacagccagaagaaaatctacgaggacgcctaccgggccgactggcaggcctacaaagaggaaatcaaccgcatccaagaacagctgacccctagccagatcctgagcctggaaaaagaaatcatgcagaagcggctgaagaagaaggccctgatcaagaaacgggtgctgaccatgctgggcaagcctaagaaacccagaagcgccttcaacatcttcgtgtccgagtgcttccaagaggccaaagaggatagccctcaggccaagatgaagacactgatcctgaactggaagaacctgcctgatcctcagaaacaggtgtacatccagctggccaaggacgacaagatcagatacgacaacgccatgaagtcctgggagaagcagatgatcgacatcggccggaaggatctgatccggcggaagatgaaaacccagcctaaggacggcaccgagaagagagtcgactga
Hedgehog TFAM	Atggctctgctgagaggcgtttggggagtgctgtctgccctgggaaaaacaggcgccgatctgtgtgccggctgtggcagcagactgagattccctctgagcttcgtgtgcctgcctcggtggtttagcagcaccctgggcagataccccaagaagcctctgagcagctacctgcggtttagcaccgagcagctgcccatgctgaaggcccagaatcctgacgccaagaacagcgagctgatcaagaagatcgcccagatttggagagagctgcccgacagcgagaagaagctgtacgaggacgcctacagagccgactgggaagcctacaaagaggaaatcaaccgcatccaagagcagctgacccctaaccagatcctgaccatggaaaaagaagtgatgcagcggcggctgaagaaaaaggccctgatgaagaaacgcgagctgagcatgctgggcaagcccaagaggcctagaagcgcctacaacatctttatcgccgagcggtttcaagaggccaaggacgaaagcagccaggccaagctgaaaaccgtgaacgagaactggaagaacctgagcagcagccagaagcaaatctacatccagctggccaaggatgacaagatccgctacgacaacgagatgaagtcttgggaagcccagatgatcgaagtgggcagaaacgacctcgtgcggagaaccgtgcctaacaagaagaaccggacaaaggacgacagcgcccaggtcgactga

Elephant TFAM	Atggctctgctgagaggcgtttggggagtgctgtctgccctgggaaaatctggcgccgagctgtgtagcagctgtggcagcagactgagattccctctgagcttcgtgtgcgtgcccagatggtttagcagcaccctgggcagctaccccaagaagcctctgagcagctacctgcggtttagcaccgagcagctgcccatcttcaaggcccagaatcctgacgccaagaacagcgagctgatcaagaaaatcgcccagctctggcgggacctgcctgactctgagaagaaggtgtacgaggacgcctacagagccgactggcaggcctacaaagaagaagtgaaccgcatccaagagcagctgacccctagccagatcgtgtctatggaaaaagaaatcatgcagaagcggctgaagcggaaggccctgatcaaaaagcgcgagctgaccatgctgggcaagcccaaaagacccagaaccgcctacaacatcttcatcagcgagagcttccaagaggccaaggacgccagctctcaggccaagatgaagaccgtgaacgagaactggaagaacctgagcagcagccagaaacaggtgtacatccagctggccaaggatgacaagatccgctacgacaacgagatgaagctgtgggaagcccagatgatcgaagtgggcagaaacgacctgatccggcggaagatgaagcagcggaccaaggatggcaccggcaacgtgaagattgtcgactga
Elephant shrew TFAM	Atggctctgctgagaggcgtttggggagcactgtctgccctgggaaaatctggcgccgaactgagagccagatacggcaacctgctgcggttccctctgagcttcctgtacatccccagatggttcagcagcaccatcggcacataccccaagaagcccctgagcagcttcctgagattcagcaccgagcagctgcccatcttcaaggcccagaatcctgacgccaagaacagcgagctggtcaagaaaatcgcccagctgtggcgcgagctgcccgacagcaagaagaaaatctacgaggacgcctaccgggccgactggcaggcctacaaagaggaaatcaaccgcatccaagagcagctgacccctagccagatcatgagcatcgagaaagagctgacccagaagcggctgaagcggaaggccctgatcaagaaacgcgagctgaccatgctgggcaagcccaagaggcctagaagcgcctacaacatcttcatcagcgagtgctttcaagaggccaaggacagcagcagccaggccaagctgaaaaccgtgaacgagagctggaagaacctgacctccagccagaaacaggtgtacatccagctggccaaggatgacaagatccgctacgacaacgagatgaagctgtgggaagcccagatgatcgaagtgggcagacacgacctgatccggcggaagatgatgccccagaccaagaacgagaacgtcgactga
Little brown bat TFAM	Atgccgcttttgaggggtgtttggggtgtcctgtcagcattggggaaatctggagctgaactttgcgctgactgtggcactcgactgtgtgcacctttcaggttcgtctacattccacgctggtttagctctaccctctcatcctaccccaagaaacccatgacctcttatgtgaggtttagcaaggaacagcttgctatttacaaagcgcgcaatcctgaagccaagaactccgagctgatcaagaaaattgcggagatttggagagaactccctgagagcgaaaagaagatttatgaggacgcctataaagccgattggcaggcatacaaagaagagctgaatcgcatccaggagcaactgacaccatcacagaaggtgtcactggagaaagagatgatgcagaagagactgaagaagaaaagcatcctgaagaaacgggaactgacactgctcggcaaaccaaaacggcccagaagtgcctacaacatcttcatcagcgaatgctttcaaggcgccaagaacgggagtagtcaggtgcgactcaaatccatacacgaaagctggaagaatctgagttctgccgaaaagcaagtgtacatacagctggcagaggatgacaaggctaggtactatagcgagatcaaatcctgggaggagcagatggttgaagtaggaagagaggatctgttgcgtcggaaggtgaaaccccagtccaagtctacggagaagtatggatcctag
Coelacanth TFAM	atgccccagccagctaggtctgtcactttcagacgaggcgctctgagaggaactggcagcgcattcctgttcatcagatcagccttggcaatggtgtccatcataagcggatgtgccaaatccttcctcaaaacacttggagggtttcgcaccagatgctctggggtgtacctgactgaacgctggtttagcgacaattccggatcacctcgtccaccaaagcgcccgattagcgcatatctccggtatgccatggaacaacagcccattctgattaagcagaatcccgatgtgaagatcgtcgagatcaccaaacagattgccttggggtggaaagaactgagtgtggcacagaaacaaccctatgaagccatcgctgacgtacacaaacagaagtacagggaggaaatggagaagtacaaagcgcagcttacaccagctcagctggctgctctgcaggaacaaagacgacaaaagatggccaagcgcaagctgattcggcgcaaacgtgagctgacgatgctcggcaagcctaaacggcctagaagtgcctttaacatctttatggcggagcatttcgaggaagccaagggtgccacaatgcaagcgaagatgaaaaccctgtttgaggattggaacaacttccacacttcccagaaacagatgtatctgcagcttgccgaagatgacaagatcaggtacgagaatgagatgaagagttgggaggagcatatgatagacatagggagggaggatctcatccgggtgaaacagcggaagaaaatgggtactgtgaaaggcaacaagagcatgaccaaggtcatttctaccaaagttaggggcaagaaggctcctagcaccaagaaaacaccggtttcacagaccaagaaaacagcacagcgaacgaagaaggcagaagagggatcctag

Zebrafish TFAM	Atggctccattcagcttgatgtcagtgggggctaatctgctggtcaagtcattcagcctgttctcgagtgcatctgttgtgaggtgttcatgt<br>gtagctccagcgataaagagttttagcactgcaactcgaggtccacccaagagacccctgacagcatacatgacatttgtaaaggatatg<br>cagcccaccgtcagcaaacaaaatcctagcataaaaagtgtggatgttatgcgaaagattgcccagcagtggaaaatgttgaccaccga<br>acaaaagcagccatttcaggttgcctctctggaggccaaggagcaatataaacttgccttggagaaattcaaagcccagctcacccctgc<br>agagagtgcggcttttgcggaggaaaaacgacaaagagtggccaaaagaaaagccattcggaaaaaaaaggaattgaataatcttgg<br>caagcccaaacgcccaaggagcaccttcaacatcttcatggcggagcattttgtggaggcaaagggaactactacgcaggcaaaattga<br>agtcactaagagatgactggaatcgcttgagcgacacacaaaaacaaatgtacatacagctggcagaggacgataaagtccgttacaa<br>gaatgaaatcaaatcgtgggaggagcacatgatggagatcggaagagaagatcttctgcggagaaagacgaagagcgccctcaaagc<br>caaagccaaaaccaaaaccatagccactaaaaacaggaaaaagacatccaaagtgacagtgataaaggcaaaagcgcccaaaaag<br>aagaaagatgctagtggaaaagctgtgtatttctaa
Xenopus TFAM	Agacagactggaagcctggccctggtcgcaagagccggacagaaagatcgggtcctgagcagcctgcccctggtcatgatgagcctgat<br>gtccaggggcgtgggaggactgctgcgctctctgaccggcatcaactgcagccagaccatcaggtgtacaaatgtgagctccggcatctt<br>cggctttcagtgcagccctctgaggtgtttctccaagggagcagcaccatctgactacccaaagaggcctctgagcggatacctgcggtat<br>tccgtggagcagagaccaaagctgcacaagcagtatcccgaggccaagatgatggacctgaccaagatcatcgccctggagtggaagg<br>gactggcaagcacagagaaggagccatacgaggcagtggcaaaggcagatctgaagaagtaccgggaggaggtgaagcagtataga<br>gaggccctgagccctgtgcagctggagctgcacagggagcagcggagacagaggctggccaagcgcaagtccgtgaggaagaagcgc<br>gagctgaccgccctgggcagaccaaagaggccacgctcccctttcaacatctttatgtctgagcacttccaggacgccaagggcacctct<br>agccagacaaagatgaagtctctgcgggatgagtgggagagactgcacaacacccagaagcagacatacaatcacctggcccaggac<br>gataagatccggtatgagaatgagatgaagagctgggaggagcagatgatcgagatcggcaggggcgatctgatccgcctgaaccagc<br>ggaagcggttcaagaagcccagagccacaagagcctcctctaagaccaatacagccaagaagggcctgacaggctcatcccacaaag<br>aggggttcagcggggcaagcaatcccaaacagcacgaagagtaa
Armadillo TFAM	Atggctctgcttagaggcgtgtggggactgctgtctagcctgggaaaatctggcgcccagctgtactccggctgcggcaatagactgaga<br>ttcagcctgagcttcgtgtgcacccctcggtggtactcttctaccctgggcagcttccccaagaagcccctgaacagctacgtcagattcgc<br>caaagagcagctgcccatcatcaaggcccagaatcctgacgccaagaacaccgagctgatcaagaaaatcgcccagctctggcgcgag<br>ctgcccgactctcagaagaaaatctacgaggacgcctacagagtggactggcaggcctacaaagaggccatcaacagaatccaagagc<br>acctgacacctagccacatcctgagcctggaaaaagagatgcagaagcggctgcccaaagaggtgctgatcaaaaagcgggtgctgac<br>catgctgggcaagcctaagaaacccagaagcgccttcaacatcttcgtgtccgagtgcttccaagaggccaaagaacacagccctcagg<br>ccaagatgaagaccatcaaccagaactggaaggacctgcctgacagccagaaacaggtgtacgtgcagctggccaaggacgacaaga<br>tcagatacgacaacgagatgaagtcctgggaagaacagatgatcgagatcggccggaacgacctgatccggcggaaaatgaagaacc<br>ctcctaaggacgccaccgagaagtgcgtcgactga

Tasmanian devil TFAM	Atgatggtggagaaggacgtgctcaatgggaacaaaatagtcctcggtgttgtgcgtgctaaggtggacctggcagcctccgttctctcccatacccgcggcatttgccaggagccttcgcctacttgggaaggcacgacagccagacctttcccggctgcatgaccctgtccatctgtggactggaatggttcatgaaagactgcacgcttagtaatgcccctaagaaacctctttccgcttacattcgattcaccatggagcatagaccctgctcaaagagcagaatcctgatttgaagagcactgaaatcattaagaaactggcagaagcgtggcgggaactgccacaaagcaagaagaaggtgtatgaagaggcaacaaaagcagagtttgaggtatacaaggaggaaaactctaaatacattgctgaactgaaccacgctgaaaagaagaatctgaaggaggagaagcgcagaaaacgggtgaggaaagagatcattaagaagaagcgcgagctgacaatctttggcaaacccaagaaacctcgctcagggtataacatcttcattagcgagcactttaaggaagggaaaggtataagttctcaggagactatgaaaattctgaacgaagagtggaagaatctgtctcccagccagaaacaggtctatcttcaactggcggaagatgataaaattagatacgccaacgaaatcaagtcatgggaggagaagatgatagaaatcggcagggaggatctgctgagattccgaaaactctcagccaagatgggcaaacatcttgaggacatctatggatcctga
MTS-Tasmanian devil TFAM	atgctgtttaatctgaggattctgttaaacaatgcagcttttagaaatggtcacaacttcatggttcgaaattttcggtgtggacaaccactacaagagcagaaactcatcagcgaggaggacctgggatccgactgcacgcttagtaatgcccctaagaaacctctttccgcttacattcgattcaccatggagcatagaccccctgctcaaagagcagaatcctgatttgaagagcactgaaatcattaagaaactggcagaagcgtggcgggaactgccacaaagcaagaagaaggtgtatgaagaggcaacaaaagcagagtttgaggtatacaaggaggaaaactctaaatacattgctgaactgaaccacgctgaaaagaagaatctgaaggaggagaagcgcagaaaacgggtgaggaaagagatcattaagaagaagcgcgagctgacaatctttggcaaacccaagaaacctcgctcagggtataacatcttcattagcgagcactttaaggaagggaaaggtataagttctcaggagactatgaaaattctgaacgaagagtggaagaatctgtctcccagccagaaacaggtctatcttcaactggcggaagatgataaaattagatacgccaacgaaatcaagtcatgggaggagaagatgatagaaatcggcagggaggatctgctgagattccgaaaactctcagccaagatgggcaaacatcttgaggacatctattga
Opossum TFAM	Atggctgctggtgctgctgcacttcttagaggcggatggcgagcactgagagccctggatagacctgctgctctgagagccgccgctggcattgatagaggactgcttggacctcctctgcctagcagcagcatctgcgccctggaacggttcatgaaggactgcaccctgagcaacgtgcccaagaagcctctgaccagctacatcagattcgtgatggacagacagccccagttcaaagagcagaaccccgacctgaagaacaccgaagtgatccggatgctggcccaagtgtggcgagaactgcctgcctctgagaagaaggtgtacgaggacgccaccaaggccgacttcaagctgtaccaagaacaggtgtccaagtacaaggccgagctgaaagtgggagagaagcggaacctgaaggtggaacggcggagaaagaaggcccggaaagaaatcgtgaagaagaaacgcgagctgaccgtgttcggcaagcccaagaggcctagaagcggctacaacatcttcatcagcgagaacttcaaagagagcagaggcctgcctgctcaagagatgctgaagatcctgaacaaagagtggaagaacctgagcagcagccggaaacaggtgtacatgcagctggccgaggacgacaagatccggtacaccaacgagatcaagagctgggaagagaagatgatcgagatcggcagagaggacctgctgagattccggaagctgaaggacaagatcggcaagggccttgaggacatctacgtcgactga
Platypus TFAM	Atgcgtagacgaggagcttgcgcacccctgagcgccgctctgtctgccggatcaggcaggacagcccctggtagtgccatggctgccgctgcggcagccttgggagccctggccagagcggcagacagaccacctctgactctgcggtgtgtactgagtaagagcttctctaaagggtcatccgttgccaaaagaccacgccgacctctgacagcttatcttcgctttctggcacaacagaggagcatcttcaagaagcaaaccccggatatgaagaatgaggagattgtgaagaaatccgcggagatgtggagggaactccctgaggtcgagaaacaggtgtatcggaaagcagcaaacgttgactgggaagccttcagagaggaaatggctaagtaccaagctcagctgactcccctccagcgcattgctatgaaaatcgagaagttggagaagcaggccaaaagtcgtgcttccaagaagaagcgggaactcaccgtgtttgggaaacccaaagaaacgaggcttccccagaacatattcgtgtctgagcactatcagggcgcaaagggtgatagctggcaggaaaagatcaagtcattgtttgaggcctggaagaatctgccatcctctcagaagcaagtctactttcagctggcccaggaagataagattcggtacgaaaacgagatgaaaatctgggagaagcagatgattgacataggccggaaagaccttctctacgtgaaaagccgcaggagcaaaatcaaagatgacgatggatcctga

MTS-Platypus TFAM	atgctgtttaatctgaggattctgttaaacaatgcagcttttagaaatggtcacaacttcatggttcgaaattttcggtgtggacaaccactacaaggatcctctaaagggtcatccgttgccaaaagaccacgccgacctctgacagcttatcttcgctttctggcacaacagaggagcatcttcaagaagcaaaccccggatatgaagaatgaggagattgtgaagaaatccgcggagatgtggagggaactccctgaggtcgagaaacaggtgtatcggaaagcagcaaacgttgactgggaagccttcagagaggaaatggctaagtaccaagctcagctgactcccctccagcgcattgctatgaaaatcgagaagttggagaagcaggccaaaagtcgtgcttccaagaagaagcgggaactcaccgtgtttgggaaacccaaagaaacgaggcttccccagaacatattcgtgtctgagcactatcagggcgcaaagggtgatagctggcaggaaaagatcaagtcattgtttgaggcctggaagaatctgccatcctctcagaagcaagtctactttcagctggcccaggaagataagattcggtacgaaaacgagatgaaaatctgggagaagcagatgattgacataggccggaaagaccttctctacgtgaaaagccgcaggagcaaaatcaaagatgacgattga
Chicken TFAM	Atggccgctgcactcgccctgctgggccgggccgcaggactggccaacggcgcccgccagttgttcagaggatgcggtatcggaagagccgcagaggcgggctgttgtagagcaatgtcctccgcagagaggcccaaaagacccctgagcgcttatttcagattcttgagggataaccagcccgcctttcgacagcaaaatcccgagctgaactcactcgaactcgtgaagaaattggcgggggtgtggcgcgagctccccgcctcccagaagcaagtgtacgaagaggctcgcaaaacagattggcgcaagtatgaggagcagctggctgcgtataaggcccaactgactcccgcccaggcagccgccttgaaagaggaaagaaggaaacggctggcgaagcggcggtccttccgaattaaacgggagctgacagtgttggggaagccgaagcgcccacgctcagggttcaacatatttgtgtccgaaaactttcagcagtccaagggccttagcccaacggccaagctcaagcagttgttcgaaacctggcagaatctgtcctcaagccagaagcaaccatacctgcagctcgcccaggacgataaggtaagataccagaacgaaatgaaatcctgggaggcgaagatggtagagctgggcagagaagaccttattcgaagcagagagcagcggcccaagaaaaagaccgacacagctcaggaaggaagcaaggcttcattgagggagtcactggccaaattgaagctgaagaaatcagaagaatga
Green sea turtle TFAM	Atggattgctacgtgatggtggtgtcccagaacaagtgcagcaccacctacagcgtggaaaagtggttcagcaagcagatcagcagcgacaaccctcctaagaggccactgaccgcctacttcagattcgtgaaggaccagcagcctatcttccgcgagcagaaccccgacgtgtccatcctggaaatcgccaagaagatcgcctacgcctggaaagaactgcccgtgtccgagaagcagacctatgaggccgctgccaaggtggaacggcaggcctacaaagaggaactggccatctacaaggcccagctgagccccgctcagatcattgccctgaaagaggaaagacggcagaagcgggccaagcggaaggccatgagaaagaaacgggaactgaccgtgctgggcaagcccaagaggcctagaaccggcttcaacatcttcatgagcgagcactttcaagaggccaagggcgtgtccgtgcaggccaagatgaagaacctgttcgaggaatggcaggacctgagcaacagccagaaacagacctacctccagctggccgaggacgacaaagtccgctacgagaacgagatgaagtcctgggaagaacagatggtggacgtgggcagagaggacctgatccggtacaagaaccggcggctgagaaagagcagagccaccaccgagaagaaaaccgtgaagaaagtgatcagcaagaagcgcgtcaagaccatcaagatccagcggaccaaggacagcagcagccctgaagtgaaggccaagctgaaaacaagcagcgaggaatga
MTS-Green sea turtle	atgctgtttaatctgaggattctgttaaacaatgcagcttttagaaatggtcacaacttcatggttcgaaattttcggtgtggacaaccactacaaggatccagcaagcagatcagcagcgacaaccctcctaagaggccactgaccgcctacttcagattcgtgaaggaccagcagcctatcttccgcgagcagaaccccgacgtgtccatcctggaaatcgccaagaagatcgcctacgcctggaaagaactgcccgtgtccgagaagcagacctatgaggccgctgccaaggtggaacggcaggcctacaaagaggaactggccatctacaaggcccagctgagccccgctcagatcattgccctgaaagaggaaagacggcagaagcgggccaagcggaaggccatgagaaagaaacgggaactgaccgtgctgggcaagcccaagaggcctagaaccggcttcaacatcttcatgagcgagcactttcaagaggccaagggcgtgtccgtgcaggccaagatgaagaacctgttcgaggaatggcaggacctgagcaacagccagaaacagacctacctccagctggccgaggacgacaaagtccgctacgagaacgagatgaagtcctgggaagaacagatggtggacgtgggcagagaggacctgatccggtacaagaaccggcggctgagaaagagcagagccaccaccgagaagaaaaccgtgaagaaagtgatcagcaagaagcgcgtcaagaccatcaagatccagcggaccaaggacagcagcagccctgaagtgaaggccaagctgaaaacaagcagcgaggaatga

Bald eagle TFAM	Atgtacggaatgcctgctggcggaggtagacctggacctggatgttttgctcctcctggaccaggtgcaggcggagtggctgctagaagaagtgtgacaggcgtcgccgtcgtgtgtagagttggaggatctgccgagaagggcctgagcagaggcatcagctctgacgagcctccaaagaggccactgaccgcctacttccggttcatgaaggaaaaccacagcgccttccggcagaaaaaccccgaagtgaccaacatggaactgatcaagaagatcgccgaggcctggaaagagctgcccgcctctcagaaacaggtgtacgaggaaagcagaaaggccgactggcagcggtacaaagaacagctggccgcctataaggcccagctgacacctgttcaagccgccgctctgaaagaggaaaagcggaagcagctggccaagcggagactgtttagagccaagcgcgagctgaccatgctgggcaagcccaaaagacccagaagcggcttcaacatcttcgtgtccgagaacttccaagagagcgagggcatcagccccatggccaagctcaagcagctgtttgatgcctggcggaagctgagcagcagccagaagcagccttatctgcaactggccgaggacgacaaagtccgctacgtgaacgagatgaagtcctgggaagccaagatggtggaactgggcagagaggacctgatccggtccagaaccagaatgcccagaacaaagaccgccgagacagccaagaaagccgccacagccaaggccagcagcagagagaacaaggccaagctgaaactgaagaagtccgaggaatag
Python TFAM	Atggctgctgctctgctggccagaatgctggcctctgtgtcagtgtctggaccccactgtgctctgagatgcggcgtgacatgcagcctggaaaagtgcttcctgaagtacatcagcagcgacagctgccccaagaggcccctgactccttacctgcactttctgaaggaccagcggcacatctaccagaagaagttccccgagctgaacagccagcagatcaccaagcagctgggccatctttggagagagctgcccgagtctgagaagcagcactacgaggccatcagcaaggccgagtgggacatcttcagagaacagatggccaagtacaagagcgagctgagccccgtgcaagaggaagccctgaaagaagagaagaaaatgaagaagcaagtccggaaagaggccaagatcaagaaagaactggtggccctgggcaagcccaagagaagcagaaacgccttcaacatcttcttcagcgagaacttccaagaagccaagggcaccagcaacaaagagaagctgacagccctgagcgaggaatggaagaacctgcctagcagccagaagcaaatctactgtcagctggccgaggacgacaagatcagatacgacaacgagcagcggagctgggagcagcagatgaaggaagccggaagagaggacgttctgaacttcaagatcacccggcggagaacccgcagaaagaccgtgacagagcctctgatcggcaagtga
Elephant shark TFAM	Atgttttctctggaacggggcagcgtgacccctcctaaaagacctatgagcgcctacctgagatacgtggtggaacagcacagagtgctgctgagacaggcccctgacctgaagatcgtggaaaagaccaagaagatcgcccaggcttggcggcacctgacacctgaacagaagcagccttacgagatcgccgccaacgagggcaagctgaagtacaaagaagaggtggccacattcaaggccaatcacacccctaccgagctggccatcttcaaagaggaaaagcggaagaagctgacccggcggagaatcatgcggcagaaaagaaagctgaccatgctgggcaagcccaagagaagcagaaccgccttcaacatcttcatgagcgagcacttcgacgaggccaagggctctacagtgcaggccaagctgaaaaacctccaggacgagtggcacagactgcccgagagccagaaaaagatgtacacccagctggccgaggacgacaagatcagataccagaacgagatcaagagctgggaagaacagatgatcgaggccggccacgaggatctcgtccggatgaagcagaagggcagaacctctgccaagcgggccgtgtccaaagtgatccctaccaaggccaagaccagcaagcccagcaccacaagcaatcccgccaagagcgtgaagtccaagaaaaaggccgaggaatga
MTS-elephant shark	atgctgtttaatctgaggattctgttaaacaatgcagcttttagaaatggtcacaacttcatggttcgaaattttcggtgtggacaaccactacaaggatccgaacggggcagcgtgacccctcctaaaagacctatgagcgcctacctgagatacgtggtggaacagcacagagtgctgctgagacaggcccctgacctgaagatcgtggaaaagaccaagaagatcgcccaggcttggcggcacctgacacctgaacagaagcagccttacgagatcgccgccaacgagggcaagctgaagtacaaagaagaggtggccacattcaaggccaatcacacccctaccgagctggccatcttcaaagaggaaaagcggaagaagctgacccggcggagaatcatgcggcagaaaagaaagctgaccatgctgggcaagcccaagagaagcagaaccgccttcaacatcttcatgagcgagcacttcgacgaggccaagggctctacagtgcaggccaagctgaaaaacctccaggacgagtggcacagactgcccgagagccagaaaaagatgtacacccagctggccgaggacgacaagatcagataccagaacgagatcaagagctgggaagaacagatgatcgaggccggccacgaggatctcgtccggatgaagcagaagggcagaacctctgccaagcgggccgtgtccaaagtgatccctaccaaggccaagaccagcaagcccagcaccacaagcaatcccgccaagagcgtgaagtccaagaaaaaggccgaggaatga

Alligator TFAM	Atggctgctacactgctcggacaggtgctggcctatgctctgcacacacagagactgctgagatgcagcgcccccttgttctggccctgccagaagatggttctgccaagagcctggacctcctagcgctcctaaacctcctgccagagccttcatccggttctacgtggaccaggccgtgaccgtgaagaagcagaaccctggcatccgggtgtccgagatggccaaaaaagtggcccacacatggcggagcctgcctgtgtctgagaagcaggcctataaggccgccgaagagatggacatgcaggtctacaaagagcagctggccctgtacaaagcccagctgacccctagccagaaagccgctctgatggaagagaggatcaagaagaaggccagcagagagctgaagcggaagaaaaaagaactggccatgttcggcaagcccaagaagccctacaacagcttcaacttcttcatggccgagcggttccaagaggccaagggaatttctgccccagccaagatcaagtggctgggcgacgaatggcagaatctgagcagcagcgagaaacagaactacgtgcagctggctgaggacgacaagatcagatacgccaacgagatgaagctgtggaaagaacagatgatcgaggctggcagagaggacctgctgtcctccgagaagcggttccggatgctgagaaacaagggcaccaagcctgtgaaggtgtccgaagtgaaaaccgtgatgatcagcaagaccgccaaggccagctctagccctgaagtgctggccaaggtggtcaagaccaagaaaagcgaggaatga
Drosophila TFAM	Atgatctacaccacaacactgatgtcctcgcgcggcggcctcatcggctcgctgatcaacaaagtcaggcccctagcagccgccagcatcagcaacactccggcggtgccgtcgaagaccctggaggagcagttgggcctgccgccgcgaccaaagaaaccgctgactccctactttcgcttcatgcgggagcagcggcccaagctgaaggctgccaatccccagattaccaccgtcgaggtggtgcgccagctgtctaagaactggtccgatgccgatgcgcagctgaaggagcgcctgcaggccgagttcaagcgggaccaacaaatctacgtggaggagcgaacaaagtacgatgccacactcacggaggagcagcgggccgagatcaagcagctcaagcaggacctcgttgacgccaaggagcgccgccagctgcgcaagcgggtcaaggagctggggcgacccaaaaagcccgcttcggccttcctgcgattcatcgccagcgaacgtatcaacactccgcagggcgacaagcaaacctaccgcgagtggcaccaaaagaccaccgccaagtggactcgcctttccgactccgagaaggaggtctacatgcaggagtcgcgcaaggagatggagctctacaggaaagcgatttccgtttgggaggagaagatgatccgcctgggccacatcgacgtggtgcgtcacggaaatcttatcgatccacctgagccaaagccccgcaagacgctggcctccaaagatatatag
Lancelet TFAM	Atggcagctgccaccaagctgagcttgggagtgcagtatctcctgtggggcaccaaatgctccatctcctttgggacaaggccgacgacacctcttctgcagtctagcagatggttcgttcagagctcaagcaaatttccgacaccacccaagcgtcctgccaatgcctacattcgctacgtcaatcagaaaatgcctactgtacggagccaaaatccaggtgcgggaccaaaacagattgtgcgcatatgtgccagtctgtggaaacagctgagtgacactgagaagcagccctatatcaacgatgcagctgcagagcgtgagaagtacaaaagcgagtatgaggatttcaaatccgacttcaccattgaggagtggggagagtatcaggactcaagaaggcaacgacggcgacaactgcagaacagaagaaggcgggaagaactgcaacagcttgggaaacccaagcaacccgttcacggctatgggctctttgtgaaggagatgttgtctggctcctcagggaacatggcccagcaaatgaaggacatgaagggtcagtggatcaatctcccagaaacggagaaacagaagtacatgtctgaagccaacaaggccaaggagaaatacgaagccgaaatgcaggcgtgggaaaagaagatggtggatcagggtaggcctgaactggtgcgcggatacaaaccacccaagaccaccgataagaagaagaaacccaagaagaaaaccatcgctactaaggctgcaaagaaaaccacaaaacggacaactgtcaaactggctggcaagaagaaacctggcacactcagtactgcacgctggcctggatcctag
Sea urchin TFAM	Atggcctctagtctgggtgcatttgctcggatcttctaccagaattgccggatttggagcgtttccactacacggcctggcttgttgggtagctcatctcagcccaaactgccttggatgcaagacagaaaggagtttagcgaggacatacccgctaagcccaaacgaccactcacatccttcttccagttcacgagcgagcagaggcccaaactgactgccatggaaccaaacctgtcagtcactgatgtgaccaagcgcataggagctatgtggcgcgatctctcagaggacgagaaggaagtgtacaggcttgactttgagtccagaagggagaaatacaccgaggaaatggaggactatcgctctcgtctgaccgatgagcagctcgatagcatgagtgagatcgatcggaacaaacgtgagatgaaggcgaaaagacgccataagagcgagatgaagaagctgaataagccgaaacgacctccaacaggctacagtctgttcatcaaggcccagtttaaccaacagccagcaggaggcagaaccagagaagagattcaagcccagtttaaagaggctgcatccatctggcattcccttcctgaccacgaaaagcagcagtatcacgaagaagcctctctgctgacggagacatatagggaagaaatggaagagtggaagaggaaaatggaaggggaagggattagcaccggatcctag

Acorn worm TFAM	Atggctctccatgctgtcgtaggtgtgcgttttctggccctcaaactctccaaacagtgttgcatttccactactctgcggtatgtggaacct<br>gtacacattcaggctgcaagatgtctgtctcagaatgaggaactgcctaggaaaccaaagcgcccaaccacacccttcgtggtgtttgcc<br>gaaaagatgttcaatagcagtcctgagcgaaactactctaagatcctgaaggatgccagcaaggaatgggccaacatgtccgatgagg<br>acaagacccagtattacgacacctacaaagagcgatttgccaagtacaaagaggagatgaaaacctatcaaccaacagaagaagaaa<br>ccgaagctcagaggaagaagaaagccagagaacagaggcgcttgacgttgaagaagctggcaaaactggggaaacccaaacttcctg<br>gaagctcatacagcatgtatgttcgggaaaagatgaagtctgcagatagtaagggacgccagaaagaggtgatgatagaggcgtcaac<br>tgactggaagttccttacagaggaggagaaacagccctacaaagacatcttcctgcaggaaaagaagcggtatatgaacgagatggag<br>gtctgggagaagaaaatgatcaagctcggcaacaatgacctggtgaggagaaagacaaaacagccggttcaaggcatcgatgtcatta<br>attgctgggaaatagagatcaacaaacaagagagactgaagatggggcaacccattggatcctag
Nematode C. elegans HMG-5 (TFAM)	Atgttgggcacaatcagtatgcggttctttgccactaaagtcgtagctcccagagcaagcgttgcagcaagcacccccaggtcccgctc<br>ggtatgaatataaacccttatgcaatgtttatcaaagaaaatttcaaagccaatacttctgacatgaagagaaccgatctgatgaaggag<br>ttgagtgggaagtggaaagcgctgtccatttcagagaaagataaatacaccgaactttccaaaaactataacgcgcaaaagttggatga<br>ctttatgaagctgagcacagaagagcaaaaaaagctggttgactcagcgaaggagaagaaagcagagagagccagcagaagacacg<br>ccaaagagagaagggaaaagagaaaacaaagtgggaggccaagtgtccccccagtgcttatgcgcttttcattaaagaaaaattgtc<br>cggcgcgggcatggagagcaaggagaaaatgaaagaggccgtggctcaatggaaggcttttacggactcccagaagaagaaatacac<br>tgacgaggctaaaaaacttaaagacgagtaccatgtagtgctccagaaatgggaagcagaacaaaaggaaaacgcggaccagggatc<br>ctga
MTS-Yeast abf2	Atgctgtttaatctgaggattctgttaaacaatgcagcttttagaaatggtcacaacttcatggttcgaaattttcggtgtggacaaccact<br>acaagagcagaaactcatcagcgaggaggacctgggatccaacagttacagcctattaactagatctttccacgaatcttctaagcccct<br>tttcaatttggctagcaccttgttgaaggcttccaagagaacgcagcttagaaatgaattgataaaacagggtcctaaaggcccacatct<br>gcttatttcttatatttgcaagaccacagaagtcaattcgttaaggaaaatccaaccttacgtcctgctgaaatcagcaagattgccggtga<br>aaagtggcaaaatttagaggctgatataaaggaaaaatacatttctgaaagaaaaaagttgtattctgaataccaaaaggcgaagaag<br>gagtttgacgaaaaacttcctccaaagaaaccagcaggacccttcattaagtatgccaatgaagttcgttcgcaagtttttgcacaacatc<br>ctgacaagtctcaattggatctaatgaaaattatcggagataaatggcaatccttggatcaaagcattaaggacaaatacatacaagagt<br>acaaaaaagctatccaagaatataatgctcgctaccctctcaactag
H-chicken chimera	Atggcgtttctccgaagcatgtggggcgtgctgagtgccctgggaaggtctggagcagagctgtgcaccggctgtggaagtcgactgcgc<br>tccccttcagttttgtgtatttaccgaggtggttttcatctgtcttggcaagttgtccaaagaaacctgtaagttcttaccttcgattttctaa<br>agaacaactacccatatttaaagctcagaacccagatgcaaaaactacagaactaattagaagaattgcccagcgttggagggaacttc<br>ctgattcaaagaaaaaaatatatcaagatgcttatagggcggagtggcaggtatataaagaagagataagcagatttaaagaacagct<br>aactccaagtcagattatgtctttggaaaaagaaatcatggacaaacatttaaaaaggaaagctatgacaaaaaaaaaagagttaaca<br>ctgcttggaaaaccgaagcgcccacgctcagggttcaacatatttgtgtccgaaaactttcagcagtccaagggccttagcccaacggcc<br>aagctcaagcagttgttcgaaacctggcagaatctgtcctcaagccagaagcaaccatacctgcagctcgcccaggacgataaggtaag<br>ataccagaacgaaatgaaatcctgggaggcgaagatggtagagctgggcagagaagaccttattcgaagcagagagcagcggcccaa<br>gaaaaagaccgacacagctcaggaaggaagcaaggcttcattgagggagtcactggccaaattgaagctgaagaaatcagaagaatg<br>a

Chicken-h chimera	Atggccgctgcactcgccctgctgggccgggccgcaggactggccaacggcgcccgccagttgttcagaggatgcggtatcggaagagccgcagaggcgggctgttgtagagcaatgtcctccgcagagaggcccaaaagacccctgagcgcttatttcagattcttgagggataaccagcccgcctttcgacagcaaaatcccgagctgaactcactcgaactcgtgaagaaattggcgggggtgtggcgcgagctccccgcctcccagaagcaagtgtacgaagaggctcgcaaaacagattggcgcaagtatgaggagcagctggctgcgtataaggcccaactgactcccgcccaggcagccgccttgaaagaggaaagaaggaaacggctggcgaagcggcggtccttccgaattaaacgggagctgacagtgttggggaagccaaaaagacctcgttcagcttataacgtttatgtagctgaaagattccaagaagctaagggtgattcaccgcaggaaaagctgaagactgtaaaggaaaactggaaaaatctgtctgactctgaaaaggaattatatattcagcatgctaaagaggacgaaactcgttatcataatgaaatgaagtcttgggaagaacaaatgattgaagttggacgaaaggatcttctacgtcgcacaataaagaaacaacgaaaatatggtgctgaggagtgttaa
Armadillo-human chimera	atggctctgcttagaggcgtgtggggactgctgtctagcctgggaaaatctggcgcccagctgtactccggctgcggcaatagactgagattcagcctgagcttcgtgtgcacccctcggtggtactcttctaccctgggcagcttccccaagaagcccctgaacagctacgtcagattcgccaaagagcagctgcccatcatcaaggcccagaatcctgacgccaagaacaccgagctgatcaagaaaatcgcccagctctggcgcgagctgcccgactctcagaagaaaatctacgaggacgcctacagagtggactggcaggcctacaaagaggccatcaacagaatccaagagcacctgacacctagccacatcctgagcctggaaaaagagatgcagaagcggctgcccaaagaggtgctgatcaaaaagcgggtgctgaccatgctgggcaagccaaaaagacctcgttcagcttataacgtttatgtagctgaaagattccaagaagctaagggtgattcaccgcaggaaaagctgaagactgtaaaggaaaactggaaaaatctgtctgactctgaaaaggaattatatattcagcatgctaaagaggacgaaactcgttatcataatgaaatgaagtcttgggaagaacaaatgattgaagttggacgaaaggatcttctacgtcgcacaataaagaaacaacgaaaatatggtgctgaggagtgttaa
Human-armadillo chimera	atggcgtttctccgaagcatgtggggcgtgctgagtgccctgggaaggtctggagcagagctgtgcaccggctgtggaagtcgactgcgctccccccttcagttttgtgtatttaccgaggtggttttcatctgtcttggcaagttgtccaaagaaacctgtaagttcttaccttcgattttctaaagaacaactacccatatttaaagctcagaacccagatgcaaaaactacagaactaattagaagaattgcccagcgttggagggaacttcctgattcaaagaaaaaaatatatcaagatgcttatagggcggagtggcaggtatataaagaagagataagcagatttaaagaacagctaactccaagtcagattatgtctttggaaaaagaaatcatggacaaacatttaaaaaggaaagctatgacaaaaaaaaaagagttaacactgcttggaaaacctaagaaacccagaagcgccttcaacatcttcgtgtccgagtgcttccaagaggccaaagaacacagccctcaggccaagatgaagaccatcaaccagaactggaaggacctgcctgacagccagaaacaggtgtacgtgcagctggccaaggacgacaagatcagatacgacaacgagatgaagtcctgggaagaacagatgatcgagatcggccggaacgacctgatccggcggaaaatgaagaaccctcctaaggacgccaccgagaagtgcgtcgactga
Tasmanian devil-human chimera	atgatggtggagaaggacgtgctcaatgggaacaaaatagtcctcggtgttgtgcgtgctaaggtggacctggcagcctccgttctctccccatacccgcggcatttgccaggagccttcgcctacttgggaaggcacgacagccagacctttcccggctgcatgaccctgtccatctgtggactggaatggttcatgaaagactgcacgcttagtaatgcccctaagaaacctctttccgcttacattcgattcaccatggagcatagaccccctgctcaaagagcagaatcctgatttgaagagcactgaaatcattaagaaactggcagaagcgtggcgggaactgccacaaagcaagaagaaggtgtatgaagaggcaacaaaagcagagtttgaggtatacaaggaggaaaactctaaatacattgctgaactgaaccacgctgaaaagaagaatctgaaggaggagaagcgcagaaaacgggtgaggaaagagatcattaagaagaagcgcgagctgacaatctttggcaaaccaaaaagacctcgttcagcttataacgtttatgtagctgaaagattccaagaagctaagggtgattcaccgcaggaaaagctgaagactgtaaaggaaaactggaaaaatctgtctgactctgaaaaggaattatatattcagcatgctaaagaggacgaaactcgttatcataatgaaatgaagtcttgggaagaacaaatgattgaagttggacgaaaggatcttctacgtcgcacaataaagaaacaacgaaaatatggtgctgaggagtgttaa

MTS-Tas. devil-human chimera	atgctgtttaatctgaggattctgttaaacaatgcagcttttagaaatggtcacaacttcatggttcgaaattttcggtgtggacaaccacta<br>caagagcagaaactcatcagcgaggaggacctgggatccgactgcacgcttagtaatgcccctaagaaacctctttccgcttacattcga<br>ttcaccatggagcatagacccctgctcaaagagcagaatcctgatttgaagagcactgaaatcattaagaaactggcagaagcgtggcg<br>ggaactgccacaaagcaagaagaaggtgtatgaagaggcaacaaaagcagagtttgaggtatacaaggaggaaaactctaaatacat<br>tgctgaactgaaccacgctgaaaagaagaatctgaaggaggagaagcgcagaaaacgggtgaggaaagagatcattaagaagaagc<br>gcgagctgacaatctttggcaaaccaaaaagacctcgttcagcttataacgtttatgtagctgaaagattccaagaagctaagggtgatt<br>caccgcaggaaaagctgaagactgtaaaggaaaactggaaaaatctgtctgactctgaaaaggaattatatattcagcatgctaaaga<br>ggacgaaactcgttatcataatgaaatgaagtcttgggaagaacaaatgattgaagttggacgaaaggatcttctacgtcgcacaataa<br>agaaacaacgaaaatatggtgctgaggagtgttaa
Human-tasmanian devil chimera	atggcgtttctccgaagcatgtggggcgtgctgagtgccctgggaaggtctggagcagagctgtgcaccggctgtggaagtcgactgcgc<br>tccccctttcagttttgtgtatttaccgaggtggttttcatctgtcttggcaagttgtccaaagaaacctgtaagttcttaccttcgattttctaa<br>agaacaactacccatatttaaagctcagaacccagatgcaaaaactacagaactaattagaagaattgcccagcgttggagggaacttc<br>ctgattcaaagaaaaaaatatatcaagatgcttatagggcggagtggcaggtatataaagaagagataagcagatttaaagaacagct<br>aactccaagtcagattatgtctttggaaaaagaaatcatggacaaacatttaaaaaggaaagctatgacaaaaaaaaaagagttaaca<br>ctgcttggaaaacccaagaaacctcgctcagggtataacatcttcattagcgagcactttaaggaagggaaaggtataagttctcaggag<br>actatgaaaattctgaacgaagagtggaagaatctgtctcccagccagaaacaggtctatcttcaactggcggaagatgataaaattag<br>atacgccaacgaaatcaagtcatgggaggagaagatgatagaaatcggcagggaggatctgctgagattccgaaaactctcagccaag<br>atgggcaaacatcttgaggacatctatggatcctga
Opossum-human chimera	Atggctgctggtgctgctgcacttcttagaggcggatggcgagcactgagagccctggatagacctgctgctctgagagccgccgctggc<br>attgatagaggactgcttggacctcctctgcctagcagcagcatctgcgccctggaacggttcatgaaggactgcaccctgagcaacgtg<br>cccaagaagcctctgaccagctacatcagattcgtgatggacagacagccccagttcaaagagcagaaccccgacctgaagaacaccg<br>aagtgatccggatgctggcccaagtgtggcgagaactgcctgcctctgagaagaaggtgtacgaggacgccaccaaggccgacttcaag<br>ctgtaccaagaacaggtgtccaagtacaaggccgagctgaaagtgggagagaagcggaacctgaaggtggaacggcggagaaagaa<br>ggcccggaaagaaatcgtgaagaagaaacgcgagctgaccgtgttcggcaagccaaaaagacctcgttcagcttataacgtttatgtag<br>ctgaaagattccaagaagctaagggtgattcaccgcaggaaaagctgaagactgtaaaggaaaactggaaaaatctgtctgactctga<br>aaaggaattatatattcagcatgctaaagaggacgaaactcgttatcataatgaaatgaagtcttgggaagaacaaatgattgaagttg<br>gacgaaaggatcttctacgtcgcacaataaagaaacaacgaaaatatggtgctgaggagtgttaa
Human-opossum chimera	atggcgtttctccgaagcatgtggggcgtgctgagtgccctgggaaggtctggagcagagctgtgcaccggctgtggaagtcgactgcgc<br>tccccctttcagttttgtgtatttaccgaggtggttttcatctgtcttggcaagttgtccaaagaaacctgtaagttcttaccttcgattttctaa<br>agaacaactacccatatttaaagctcagaacccagatgcaaaaactacagaactaattagaagaattgcccagcgttggagggaacttc<br>ctgattcaaagaaaaaaatatatcaagatgcttatagggcggagtggcaggtatataaagaagagataagcagatttaaagaacagct<br>aactccaagtcagattatgtctttggaaaaagaaatcatggacaaacatttaaaaaggaaagctatgacaaaaaaaaaagagttaaca<br>ctgcttggaaaacccaagaggcctagaagcggctacaacatcttcatcagcgagaacttcaaagagagcagaggcctgcctgctcaaga<br>gatgctgaagatcctgaacaaagagtggaagaacctgagcagcagccggaaacaggtgtacatgcagctggccgaggacgacaagat<br>ccggtacaccaacgagatcaagagctgggaagagaagatgatcgagatcggcagagaggacctgctgagattccggaagctgaagga<br>caagatcggcaagggccttgaggacatctacgtcgactga

Platypus-human chimera	atgcgtagacgaggagcttgcgcacccctgagcgccgctctgtctgccggatcaggcaggacagcccctggtagtgccatggctgccgctgcggcagccttgggagccctggccagagcggcagacagaccacctctgactctgcggtgtgtactgagtaagagcttctctaaagggtcatccgttgccaaaagaccacgccgacctctgacagcttatcttcgctttctggcacaacagaggagcatcttcaagaagcaaaccccggatatgaagaatgaggagattgtgaagaaatccgcggagatgtggagggaactccctgaggtcgagaaacaggtgtatcggaaagcagcaaacgttgactgggaagccttcagagaggaaatggctaagtaccaagctcagctgactcccctccagcgcattgctatgaaaatcgagaagttggagaagcaggccaaaagtcgtgcttccaagaagaagcgggaactcaccgtgtttgggaaaccaaaaagacctcgttcagcttataacgtttatgtagctgaaagattccaagaagctaagggtgattcaccgcaggaaaagctgaagactgtaaaggaaaactggaaaaatctgtctgactctgaaaaggaattatatattcagcatgctaaagaggacgaaactcgttatcataatgaaatgaagtcttgggaagaacaaatgattgaagttggacgaaaggatcttctacgtcgcacaataaagaaacaacgaaaatatggtgctgaggagtgttaa
MTS-Platypus-human chimera	atgctgtttaatctgaggattctgttaaacaatgcagcttttagaaatggtcacaacttcatggttcgaaattttcggtgtggacaaccactacaagagcagaaactcatcagcgaggaggacctgggatccttctctaaagggtcatccgttgccaaaagaccacgccgacctctgacagcttatcttcgctttctggcacaacagaggagcatcttcaagaagcaaaccccggatatgaagaatgaggagattgtgaagaaatccgcggagatgtggagggaactccctgaggtcgagaaacaggtgtatcggaaagcagcaaacgttgactgggaagccttcagagaggaaatggctaagtaccaagctcagctgactcccctccagcgcattgctatgaaaatcgagaagttggagaagcaggccaaaagtcgtgcttccaagaagaagcgggaactcaccgtgtttgggaaaccaaaaagacctcgttcagcttataacgtttatgtagctgaaagattccaagaagctaagggtgattcaccgcaggaaaagctgaagactgtaaaggaaaactggaaaaatctgtctgactctgaaaaggaattatatattcagcatgctaaagaggacgaaactcgttatcataatgaaatgaagtcttgggaagaacaaatgattgaagttggacgaaaggatcttctacgtcgcacaataaagaaacaacgaaaatatggtgctgaggagtgttaa
Human-platypus chimera	atggcgtttctccgaagcatgtggggcgtgctgagtgccctgggaaggtctggagcagagctgtgcaccggctgtggaagtcgactgcgctccccccttcagttttgtgtatttaccgaggtggttttcatctgtcttggcaagttgtccaaagaaacctgtaagttcttaccttcgattttctaaagaacaactacccatatttaaagctcagaacccagatgcaaaaactacagaactaattagaagaattgcccagcgttggagggaacttcctgattcaaagaaaaaaatatatcaagatgcttatagggcggagtggcaggtatataaagaagagataagcagatttaaagaacagctaactccaagtcagattatgtctttggaaaaagaaatcatggacaaacatttaaaaaggaaagctatgacaaaaaaaaaagagttaacactgcttggaaaacccaaagaaacgaggcttccccagaacatattcgtgtctgagcactatcagggcgcaaagggtgatagctggcaggaaaagatcaagtcattgtttgaggcctggaagaatctgccatcctctcagaagcaagtctactttcagctggcccaggaagataagattcggtacgaaaacgagatgaaaatctgggagaagcagatgattgacataggccggaaagaccttctctacgtgaaaagccgcaggagcaaaatcaaagatgacgattga
Green sea turtle-human chimera	atggattgctacgtgatggtggtgtcccagaacaagtgcagcaccacctacagcgtggaaaagtggttcagcaagcagatcagcagcgacaaccctcctaagaggccactgaccgcctacttcagattcgtgaaggaccagcagcctatcttccgcgagcagaaccccgacgtgtccatcctggaaatcgccaagaagatcgcctacgcctggaaagaactgcccgtgtccgagaagcagacctatgaggccgctgccaaggtggaacggcaggcctacaaagaggaactggccatctacaaggcccagctgagccccgctcagatcattgccctgaaagaggaaagacggcagaagcgggccaagcggaaggccatgagaaagaaacgggaactgaccgtgctgggcaagccaaaaagacctcgttcagcttataacgtttatgtagctgaaagattccaagaagctaagggtgattcaccgcaggaaaagctgaagactgtaaaggaaaactggaaaaatctgtctgactctgaaaaggaattatatattcagcatgctaaagaggacgaaactcgttatcataatgaaatgaagtcttgggaagaacaaatgattgaagttggacgaaaggatcttctacgtcgcacaataaagaaacaacgaaaatatggtgctgaggagtgttaa

MTS-Green sea turtle-human chimera	atgctgtttaatctgaggattctgttaaacaatgcagcttttagaaatggtcacaacttcatggttcgaaattttcggtgtggacaaccactacaagagcagaaactcatcagcgaggaggacctgggatccagcaagcagatcagcagcgacaaccctcctaagaggccactgaccgcctacttcagattcgtgaaggaccagcagcctatcttccgcgagcagaaccccgacgtgtccatcctggaaatcgccaagaagatcgcctacgcctggaaagaactgcccgtgtccgagaagcagacctatgaggccgctgccaaggtggaacggcaggcctacaaagaggaactggccatctacaaggcccagctgagccccgctcagatcattgccctgaaagaggaaagacggcagaagcgggccaagcggaaggccatgagaaagaaacgggaactgaccgtgctgggcaagccaaaaagacctcgttcagcttataacgtttatgtagctgaaagattccaagaagctaagggtgattcaccgcaggaaaagctgaagactgtaaaggaaaactggaaaaatctgtctgactctgaaaaggaattatatattcagcatgctaaagaggacgaaactcgttatcataatgaaatgaagtcttgggaagaacaaatgattgaagttggacgaaaggatcttctacgtcgcacaataaagaaacaacgaaaatatggtgctgaggagtgttaa
Human-green sea turtle chimera	atggcgtttctccgaagcatgtggggcgtgctgagtgccctgggaaggtctggagcagagctgtgcaccggctgtggaagtcgactgcgctccccccttcagttttgtgtatttaccgaggtggttttcatctgtcttggcaagttgtccaaagaaacctgtaagttcttaccttcgattttctaaagaacaactacccatatttaaagctcagaacccagatgcaaaaactacagaactaattagaagaattgcccagcgttggagggaacttcctgattcaaagaaaaaaatatatcaagatgcttatagggcggagtggcaggtatataaagaagagataagcagatttaaagaacagctaactccaagtcagattatgtctttggaaaaagaaatcatggacaaacatttaaaaaggaaagctatgacaaaaaaaaaagagttaacactgcttggaaaacccaagaggcctagaaccggcttcaacatcttcatgagcgagcactttcaagaggccaagggcgtgtccgtgcaggccaagatgaagaacctgttcgaggaatggcaggacctgagcaacagccagaaacagacctacctccagctggccgaggacgacaaagtccgctacgagaacgagatgaagtcctgggaagaacagatggtggacgtgggcagagaggacctgatccggtacaagaaccggcggctgagaaagagcagagccaccaccgagaagaaaaccgtgaagaaagtgatcagcaagaagcgcgtcaagaccatcaagatccagcggaccaaggacagcagcagccctgaagtgaaggccaagctgaaaacaagcagcgaggaatga
Python-human chimera	atggctgctgctctgctggccagaatgctggcctctgtgtcagtgtctggaccccactgtgctctgagatgcggcgtgacatgcagcctggaaaagtgcttcctgaagtacatcagcagcgacagctgccccaagaggcccctgactccttacctgcactttctgaaggaccagcggcacatctaccagaagaagttccccgagctgaacagccagcagatcaccaagcagctgggccatctttggagagagctgcccgagtctgagaagcagcactacgaggccatcagcaaggccgagtgggacatcttcagagaacagatggccaagtacaagagcgagctgagccccgtgcaagaggaagccctgaaagaagagaagaaaatgaagaagcaagtccggaaagaggccaagatcaagaaagaactggtggccctgggcaagccaaaaagacctcgttcagcttataacgtttatgtagctgaaagattccaagaagctaagggtgattcaccgcaggaaaagctgaagactgtaaaggaaaactggaaaaatctgtctgactctgaaaaggaattatatattcagcatgctaaagaggacgaaactcgttatcataatgaaatgaagtcttgggaagaacaaatgattgaagttggacgaaaggatcttctacgtcgcacaataaagaaacaacgaaaatatggtgctgaggagtgttaa
Human-python chimera	atggcgtttctccgaagcatgtggggcgtgctgagtgccctgggaaggtctggagcagagctgtgcaccggctgtggaagtcgactgcgctccccccttcagttttgtgtatttaccgaggtggttttcatctgtcttggcaagttgtccaaagaaacctgtaagttcttaccttcgattttctaaagaacaactacccatatttaaagctcagaacccagatgcaaaaactacagaactaattagaagaattgcccagcgttggagggaacttcctgattcaaagaaaaaaatatatcaagatgcttatagggcggagtggcaggtatataaagaagagataagcagatttaaagaacagctaactccaagtcagattatgtctttggaaaaagaaatcatggacaaacatttaaaaaggaaagctatgacaaaaaaaaaagagttaacactgcttggaaaacccaagagaagcagaaacgccttcaacatcttcttcagcgagaacttccaagaagccaagggcaccagcaacaaagagaagctgacagccctgagcgaggaatggaagaacctgcctagcagccagaagcaaatctactgtcagctggccgaggacgacaagatcagatacgacaacgagcagcggagctgggagcagcagatgaaggaagccggaagagaggacgttctgaacttcaagatcacccggcggagaacccgcagaaagaccgtgacagagcctctgatcggcaagtga

Elephant shark-human chimera	atgttttctctggaacggggcagcgtgacccctcctaaaagacctatgagcgcctacctgagatacgtggtggaacagcacagagtgctg<br>ctgagacaggcccctgacctgaagatcgtggaaaagaccaagaagatcgcccaggcttggcggcacctgacacctgaacagaagcagc<br>cttacgagatcgccgccaacgagggcaagctgaagtacaaagaagaggtggccacattcaaggccaatcacacccctaccgagctggc<br>catcttcaaagaggaaaagcggaagaagctgacccggcggagaatcatgcggcagaaaagaaagctgaccatgctgggcaagccaa<br>aaagacctcgttcagcttataacgtttatgtagctgaaagattccaagaagctaagggtgattcaccgcaggaaaagctgaagactgtaa<br>aggaaaactggaaaaatctgtctgactctgaaaaggaattatatattcagcatgctaaagaggacgaaactcgttatcataatgaaatg<br>aagtcttgggaagaacaaatgattgaagttggacgaaaggatcttctacgtcgcacaataaagaaacaacgaaaatatggtgctgagg<br>agtgttaa
MTS-elephant shark-human chimera	atgctgtttaatctgaggattctgttaaacaatgcagcttttagaaatggtcacaacttcatggttcgaaattttcggtgtggacaaccacta<br>caagagcagaaactcatcagcgaggaggacctgggatccgaacggggcagcgtgacccctcctaaaagacctatgagcgcctacctga<br>gatacgtggtggaacagcacagagtgctgctgagacaggcccctgacctgaagatcgtggaaaagaccaagaagatcgcccaggcttg<br>gcggcacctgacacctgaacagaagcagccttacgagatcgccgccaacgagggcaagctgaagtacaaagaagaggtggccacatt<br>caaggccaatcacacccctaccgagctggccatcttcaaagaggaaaagcggaagaagctgacccggcggagaatcatgcggcagaa<br>aagaaagctgaccatgctgggcaagccaaaaagacctcgttcagcttataacgtttatgtagctgaaagattccaagaagctaagggtg<br>attcaccgcaggaaaagctgaagactgtaaaggaaaactggaaaaatctgtctgactctgaaaaggaattatatattcagcatgctaaa<br>gaggacgaaactcgttatcataatgaaatgaagtcttgggaagaacaaatgattgaagttggacgaaaggatcttctacgtcgcacaat<br>aaagaaacaacgaaaatatggtgctgaggagtgttaa
Human-elephant shark chimera	atggcgtttctccgaagcatgtggggcgtgctgagtgccctgggaaggtctggagcagagctgtgcaccggctgtggaagtcgactgcgc<br>tccccccttcagttttgtgtatttaccgaggtggttttcatctgtcttggcaagttgtccaaagaaacctgtaagttcttaccttcgattttctaa<br>agaacaactacccatatttaaagctcagaacccagatgcaaaaactacagaactaattagaagaattgcccagcgttggagggaacttc<br>ctgattcaaagaaaaaaatatatcaagatgcttatagggcggagtggcaggtatataaagaagagataagcagatttaaagaacagct<br>aactccaagtcagattatgtctttggaaaaagaaatcatggacaaacatttaaaaaggaaagctatgacaaaaaaaaaagagttaaca<br>ctgcttggaaaacccaagagaagcagaaccgccttcaacatcttcatgagcgagcacttcgacgaggccaagggctctacagtgcaggc<br>caagctgaaaaacctccaggacgagtggcacagactgcccgagagccagaaaaagatgtacacccagctggccgaggacgacaagat<br>cagataccagaacgagatcaagagctgggaagaacagatgatcgaggccggccacgaggatctcgtccggatgaagcagaagggcag<br>aacctctgccaagcgggccgtgtccaaagtgatccctaccaaggccaagaccagcaagcccagcaccacaagcaatcccgccaagagc<br>gtgaagtccaagaaaaaggccgaggaatga
Drosophila-human chimera	atgatctacaccacaacactgatgtcctcgcgcggcggcctcatcggctcgctgatcaacaaagtcaggcccctagcagccgccagcatc<br>agcaacactccggcggtgccgtcgaagaccctggaggagcagttgggcctgccgccgcgaccaaagaaaccgctgactccctactttcg<br>cttcatgcgggagcagcggcccaagctgaaggctgccaatccccagattaccaccgtcgaggtggtgcgccagctgtctaagaactggtc<br>cgatgccgatgcgcagctgaaggagcgcctgcaggccgagttcaagcgggaccaacaaatctacgtggaggagcgaacaaagtacga<br>tgccacactcacggaggagcagcgggccgagatcaagcagctcaagcaggacctcgttgacgccaaggagcgccgccagctgcgcaa<br>gcgggtcaaggagctggggcgaccaaaaagacctcgttcagcttataacgtttatgtagctgaaagattccaagaagctaagggtgattc<br>accgcaggaaaagctgaagactgtaaaggaaaactggaaaaatctgtctgactctgaaaaggaattatatattcagcatgctaaagag<br>gacgaaactcgttatcataatgaaatgaagtcttgggaagaacaaatgattgaagttggacgaaaggatcttctacgtcgcacaataaa<br>gaaacaacgaaaatatggtgctgaggagtgttaa

Human-drosophila chimera	atggcgtttctccgaagcatgtggggcgtgctgagtgccctgggaaggtctggagcagagctgtgcaccggctgtggaagtcgactgcgctccccttcagttttgtgtatttaccgaggtggttttcatctgtcttggcaagttgtccaaagaaacctgtaagttcttaccttcgattttctaaagaacaactacccatatttaaagctcagaacccagatgcaaaaactacagaactaattagaagaattgcccagcgttggagggaacttcctgattcaaagaaaaaaatatatcaagatgcttatagggcggagtggcaggtatataaagaagagataagcagatttaaagaacagctaactccaagtcagattatgtctttggaaaaagaaatcatggacaaacatttaaaaaggaaagctatgacaaaaaaaaaagagttaacactgcttggaaaacccaaaaagcccgcttcggccttcctgcgattcatcgccagcgaacgtatcaacactccgcagggcgacaagcaaacctaccgcgagtggcaccaaaagaccaccgccaagtggactcgcctttccgactccgagaaggaggtctacatgcaggagtcgcgcaaggagatggagctctacaggaaagcgatttccgtttgggaggagaagatgatccgcctgggccacatcgacgtggtgcgtcacggaaatcttatcgatccacctgagccaaagccccgcaagacgctggcctccaaagatatatga
Lancelet-human chimera	atggcagctgccaccaagctgagcttgggagtgcagtatctcctgtggggcaccaaatgctccatctcctttgggacaaggccgacgacacctcttctgcagtctagcagatggttcgttcagagctcaagcaaatttccgacaccacccaagcgtcctgccaatgcctacattcgctacgtcaatcagaaaatgcctactgtacggagccaaaatccaggtgcgggaccaaaacagattgtgcgcatatgtgccagtctgtggaaacagctgagtgacactgagaagcagccctatatcaacgatgcagctgcagagcgtgagaagtacaaaagcgagtatgaggatttcaaatccgacttcaccattgaggagtggggagagtatcaggactcaagaaggcaacgacggcgacaactgcagaacagaagaaggcgggaagaactgcaacagcttgggaaaccaaaaagacctcgttcagcttataacgtttatgtagctgaaagattccaagaagctaagggtgattcaccgcaggaaaagctgaagactgtaaaggaaaactggaaaaatctgtctgactctgaaaaggaattatatattcagcatgctaaagaggacgaaactcgttatcataatgaaatgaagtcttgggaagaacaaatgattgaagttggacgaaaggatcttctacgtcgcacaataaagaaacaacgaaaatatggtgctgaggagtgttaa
Human-lancelet chimera	atggcgtttctccgaagcatgtggggcgtgctgagtgccctgggaaggtctggagcagagctgtgcaccggctgtggaagtcgactgcgctccccttcagttttgtgtatttaccgaggtggttttcatctgtcttggcaagttgtccaaagaaacctgtaagttcttaccttcgattttctaaagaacaactacccatatttaaagctcagaacccagatgcaaaaactacagaactaattagaagaattgcccagcgttggagggaacttcctgattcaaagaaaaaaatatatcaagatgcttatagggcggagtggcaggtatataaagaagagataagcagatttaaagaacagctaactccaagtcagattatgtctttggaaaaagaaatcatggacaaacatttaaaaaggaaagctatgacaaaaaaaaaagagttaacactgcttggaaaacccaagcaacccgttcacggctatgggctctttgtgaaggagatgttgtctggctcctcagggaacatggcccagcaaatgaaggacatgaagggtcagtggatcaatctcccagaaacggagaaacagaagtacatgtctgaagccaacaaggccaaggagaaatacgaagccgaaatgcaggcgtgggaaaagaagatggtggatcagggtaggcctgaactggtgcgcggatacaaaccacccaagaccaccgataagaagaagaaacccaagaagaaaaccatcgctactaaggctgcaaagaaaaccacaaaacggacaactgtcaaactggctggcaagaagaaacctggcacactcagtactgcacgctggccttaa
Sea urchin-human chimera	atggcctctagtctgggtgcatttgctcggatcttctaccagaattgccggatttggagcgtttccactacacggcctggcttgttgggtagctcatctcagcccaaactgccttggatgcaagacagaaaggagtttagcgaggacatacccgctaagcccaaacgaccactcacatccttcttccagttcacgagcgagcagaggcccaaactgactgccatggaaccaaacctgtcagtcactgatgtgaccaagcgcataggagctatgtggcgcgatctctcagaggacgagaaggaagtgtacaggcttgactttgagtccagaagggagaaatacaccgaggaaatggaggactatcgctctcgtctgaccgatgagcagctcgatagcatgagtgagatcgatcggaacaaacgtgagatgaaggcgaaaagacgccataagagcgagatgaagaagctgaataagccaaaaagacctcgttcagcttataacgtttatgtagctgaaagattccaagaagctaagggtgattcaccgcaggaaaagctgaagactgtaaaggaaaactggaaaaatctgtctgactctgaaaaggaattatatattcagcatgctaaagaggacgaaactcgttatcataatgaaatgaagtcttgggaagaacaaatgattgaagttggacgaaaggatcttctacgtcgcacaataaagaaacaacgaaaatatggtgctgaggagtgttaa

Human-sea urchin chimera	atggcgtttctccgaagcatgtggggcgtgctgagtgccctgggaaggtctggagcagagctgtgcaccggctgtggaagtcgactgcgc<br>tccccccttcagttttgtgtatttaccgaggtggttttcatctgtcttggcaagttgtccaaagaaacctgtaagttcttaccttcgattttctaa<br>agaacaactacccatatttaaagctcagaacccagatgcaaaaactacagaactaattagaagaattgcccagcgttggagggaacttc<br>ctgattcaaagaaaaaaatatatcaagatgcttatagggcggagtggcaggtatataaagaagagataagcagatttaaagaacagct<br>aactccaagtcagattatgtctttggaaaaagaaatcatggacaaacatttaaaaaggaaagctatgacaaaaaaaaaagagttaaca<br>ctgcttggaaaaccgaaacgacctccaacaggctacagtctgttcatcaaggcccagtttaaccaacagccagcaggaggcagaaccag<br>agaagagattcaagcccagtttaaagaggctgcatccatctggcattcccttcctgaccacgaaaagcagcagtatcacgaagaagcct<br>ctctgctgacggagacatatagggaagaaatggaagagtggaagaggaaaatggaaggggaagggattagcacctaa
Alligator-human chimera	atggctgctacactgctcggacaggtgctggcctatgctctgcacacacagagactgctgagatgcagcgccccttgttctggccctgcca<br>gaagatggttctgccaagagcctggacctcctagcgctcctaaacctcctgccagagccttcatccggttctacgtggaccaggccgtgac<br>cgtgaagaagcagaaccctggcatccgggtgtccgagatggccaaaaaagtggcccacacatggcggagcctgcctgtgtctgagaag<br>caggcctataaggccgccgaagagatggacatgcaggtctacaaagagcagctggccctgtacaaagcccagctgacccctagccaga<br>aagccgctctgatggaagagaggatcaagaagaaggccagcagagagctgaagcggaagaaaaaagaactggccatgttcggcaag<br>ccaaaaagacctcgttcagcttataacgtttatgtagctgaaagattccaagaagctaagggtgattcaccgcaggaaaagctgaagact<br>gtaaaggaaaactggaaaaatctgtctgactctgaaaaggaattatatattcagcatgctaaagaggacgaaactcgttatcataatga<br>aatgaagtcttgggaagaacaaatgattgaagttggacgaaaggatcttctacgtcgcacaataaagaaacaacgaaaatatggtgctg<br>aggagtgttaa
Human-alligator chimera	atggcgtttctccgaagcatgtggggcgtgctgagtgccctgggaaggtctggagcagagctgtgcaccggctgtggaagtcgactgcgc<br>tccccccttcagttttgtgtatttaccgaggtggttttcatctgtcttggcaagttgtccaaagaaacctgtaagttcttaccttcgattttctaa<br>agaacaactacccatatttaaagctcagaacccagatgcaaaaactacagaactaattagaagaattgcccagcgttggagggaacttc<br>ctgattcaaagaaaaaaatatatcaagatgcttatagggcggagtggcaggtatataaagaagagataagcagatttaaagaacagct<br>aactccaagtcagattatgtctttggaaaaagaaatcatggacaaacatttaaaaaggaaagctatgacaaaaaaaaaagagttaaca<br>ctgcttggaaaacccaagaagccctacaacagcttcaacttcttcatggccgagcggttccaagaggccaagggaatttctgccccagcc<br>aagatcaagtggctgggcgacgaatggcagaatctgagcagcagcgagaaacagaactacgtgcagctggctgaggacgacaagatc<br>agatacgccaacgagatgaagctgtggaaagaacagatgatcgaggctggcagagaggacctgctgtcctccgagaagcggttccgga<br>tgctgagaaacaagggcaccaagcctgtgaaggtgtccgaagtgaaaaccgtgatgatcagcaagaccgccaaggccagctctagccc<br>tgaagtgctggccaaggtggtcaagaccaagaaaagcgaggaatga
Acorn worm-human chimera	atggctctccatgctgtcgtaggtgtgcgttttctggccctcaaactctccaaacagtgttgcatttccactactctgcggtatgtggaacctg<br>tacacattcaggctgcaagatgtctgtctcagaatgaggaactgcctaggaaaccaaagcgcccaaccacacccttcgtggtgtttgccg<br>aaaagatgttcaatagcagtcctgagcgaaactactctaagatcctgaaggatgccagcaaggaatgggccaacatgtccgatgaggac<br>aagacccagtattacgacacctacaaagagcgatttgccaagtacaaagaggagatgaaaacctatcaaccaacagaagaagaaacc<br>gaagctcagaggaagaagaaagccagagaacagaggcgcttgacgttgaagaagctggcaaaactggggaaaccaaaaagacctcg<br>ttcagcttataacgtttatgtagctgaaagattccaagaagctaagggtgattcaccgcaggaaaagctgaagactgtaaaggaaaact<br>ggaaaaatctgtctgactctgaaaaggaattatatattcagcatgctaaagaggacgaaactcgttatcataatgaaatgaagtcttggg<br>aagaacaaatgattgaagttggacgaaaggatcttctacgtcgcacaataaagaaacaacgaaaatatggtgctgaggagtgttaa

Human-acorn worm chimera	atggcgtttctccgaagcatgtggggcgtgctgagtgccctgggaaggtctggagcagagctgtgcaccggctgtggaagtcgactgcgc<br>tccccccttcagttttgtgtatttaccgaggtggttttcatctgtcttggcaagttgtccaaagaaacctgtaagttcttaccttcgattttctaa<br>agaacaactacccatatttaaagctcagaacccagatgcaaaaactacagaactaattagaagaattgcccagcgttggagggaacttc<br>ctgattcaaagaaaaaaatatatcaagatgcttatagggcggagtggcaggtatataaagaagagataagcagatttaaagaacagct<br>aactccaagtcagattatgtctttggaaaaagaaatcatggacaaacatttaaaaaggaaagctatgacaaaaaaaaaagagttaaca<br>ctgcttggaaaacccaaacttcctggaagctcatacagcatgtatgttcgggaaaagatgaagtctgcagatagtaagggacgccagaa<br>agaggtgatgatagaggcgtcaactgactggaagttccttacagaggaggagaaacagccctacaaagacatcttcctgcaggaaaag<br>aagcggtatatgaacgagatggaggtctgggagaagaaaatgatcaagctcggcaacaatgacctggtgaggagaaagacaaaacag<br>ccggttcaaggcatcgatgtcattaattgctgggaaatagagatcaacaaacaagagagactgaagatggggcaacccatttaa
Nematode (C. elegans)-human chimera	atgttgggcacaatcagtatgcggttctttgccactaaagtcgtagctcccagagcaagcgttgcagcaagcacccccccaggtcccgctcg<br>gtatgaatataaacccttatgcaatgtttatcaaagaaaatttcaaagccaatacttctgacatgaagagaaccgatctgatgaaggagtt<br>gagtgggaagtggaaagcgctgtccatttcagagaaagataaatacaccgaactttccaaaaactataacgcgcaaaagttggatgact<br>ttatgaagctgagcacagaagagcaaaaaaagctggttgactcagcgaaggagaagaaagcagagagagccagcagaagacacgcc<br>aaagagagaagggaaaagagaaaacaaagtgggaggccaaaaagacctcgttcagcttataacgtttatgtagctgaaagattccaa<br>gaagctaagggtgattcaccgcaggaaaagctgaagactgtaaaggaaaactggaaaaatctgtctgactctgaaaaggaattatatat<br>tcagcatgctaaagaggacgaaactcgttatcataatgaaatgaagtcttgggaagaacaaatgattgaagttggacgaaaggatcttct<br>acgtcgcacaataaagaaacaacgaaaatatggtgctgaggagtgtta
Human-nematode (C. elegans) chimera	atggcgtttctccgaagcatgtggggcgtgctgagtgccctgggaaggtctggagcagagctgtgcaccggctgtggaagtcgactgcgc<br>tccccccttcagttttgtgtatttaccgaggtggttttcatctgtcttggcaagttgtccaaagaaacctgtaagttcttaccttcgattttctaa<br>agaacaactacccatatttaaagctcagaacccagatgcaaaaactacagaactaattagaagaattgcccagcgttggagggaacttc<br>ctgattcaaagaaaaaaatatatcaagatgcttatagggcggagtggcaggtatataaagaagagataagcagatttaaagaacagct<br>aactccaagtcagattatgtctttggaaaaagaaatcatggacaaacatttaaaaaggaaagctatgacaaaaaaaaaagagttaaca<br>ctgcttggaaaaccaagtgtcccccccagtgcttatgcgcttttcattaaagaaaaattgtccggcgcgggcatggagagcaaggagaaa<br>atgaaagaggccgtggctcaatggaaggcttttacggactcccagaagaagaaatacactgacgaggctaaaaacttaaagacgagt<br>accatgtagtgctccagaaatgggaagcagaacaaaaggaaaacgcggaccagtga

Table S3. Proficiency of chimeric TFAMs in replicating hmtDNA.

Construct	hmtDNA replication	Structural differences in mtDNA organization and in-dels in TFAM
Armadillo-human	Yes	No major changes in tRNA or ORF order. 1 aa deletion in the linker
Human-armadillo	Yes	
Opossum-human	Yes	tRNA order change around OriL from WAN-ori-CY to ACW-ori-NY
Human-opossum	Yes	
Human-platypus	Yes	No major changes
MTS-Platypus-human	Yes	
Python-human	Yes	Two control regions
Human-python	Yes	
MTS-Tasmanian devil-human	Yes	tRNA order change around OriL from WAN-ori-CY to ACW-ori-NY
Human-tasmanian devil	Yes	
MTS-Turtle-human	Yes	No major changes
Human-turtle	Yes	
Lancelet-human	Yes	No control region, altered order of tRNA genes; 1 aa deletion in HMG2
Human-lancelet	No	
Drosophila-human	Yes	Major changes in tRNA and ORF; 3 aa insertions in HMG2
Human-drosophila	No	
Sea urchin-Human	Yes	No control region; major alterations in ORF order, rRNAs separated; 5 aa insertion in HMG2
Human-sea urchin	No	
Nematode-human	No	466 bp AT-rich control region flanked by MT-TA and MT-TP instead of MT-TP and MT-TF, altered tRNA gene order, rRNA genes are widely separated. 1 aa insertion in HMG1, 1 aa deletion in HMG2
Human-nematode	No	
Alligator-human	No	MT-TF moved to MT-TP on the left side of the D-loop; MT-TS2 and MT-TH swapped
Human-alligator	No	
Acorn worm-human	No	MT-ND1 and MT-ND2 swapped, MT-CYB and MT-ND6 swapped, control region between MT-ATP8 and MT-ATP6. 4aa deletion HMG1, 6 aa deletion link, 1 aa insertion HMG2
Human-acorn worm	No	
